# The impact of molecular variants, crystallization conditions and the space group on ligand–protein complexes: a case study on bacterial phosphotriesterase

**DOI:** 10.1107/S2059798323007672

**Published:** 2023-10-20

**Authors:** Orly Dym, Nidhi Aggarwal, Yacov Ashani, Haim Leader, Shira Albeck, Tamar Unger, Shelly Hamer-Rogotner, Israel Silman, Dan S. Tawfik, Joel L. Sussman

**Affiliations:** a Department of Life Sciences Core Facilities, Weizmann Institute of Science, Rehovot, Israel; b Department of Biomolecular Sciences, Weizmann Institute of Science, Rehovot, Israel; c Department of Brain Sciences, Weizmann Institute of Science, Rehovot, Israel; d Department of Chemical and Structural Biology, Weizmann Institute of Science, Rehovot, Israel; UNSW Sydney, Australia

**Keywords:** crystallization conditions, zinc ions, phosphotriesterases, organophosphates, space groups, bioscavengers, expression systems

## Abstract

A bacterial phosphotriesterase was employed as an experimental paradigm to examine the effects of multiple factors, such as the molecular constructs, the ligands used during protein expression and purification, the crystallization conditions and the space group, on the visualization of molecular complexes of ligands with a target enzyme. In this case, the ligands used were organophos­phates that are fragments of the nerve agents and insecticides on which the enzyme acts as a bioscavenger. 12 crystal structures of various phospho­triesterase constructs obtained by directed evolution were analyzed, with resolutions of up to 1.38 Å. Both apo forms and holo forms, complexed with the organophosphate ligands, were studied. Crystals obtained from three different crystallization conditions, crystallized in four space groups, with and without N-terminal tags, were utilized to investigate the impact of these factors on visualizing the organophosphate complexes of the enzyme. The study revealed that the tags used for protein expression can lodge in the active site and hinder ligand binding. Furthermore, the space group in which the protein crystallizes can significantly impact the visualization of bound ligands. It was also observed that the crystallization precipitants can compete with, and even preclude, ligand binding, leading to false positives or to the incorrect identification of lead drug candidates. One of the co-crystallization conditions enabled the definition of the spaces that accommodate the substituents attached to the P atom of several products of organophosphate substrates after detachment of the leaving group. The crystal structures of the complexes of phosphotriesterase with the organophosphate products reveal similar short interaction distances of the two partially charged O atoms of the P—O bonds with the exposed β-Zn^2+^ ion and the buried α-Zn^2+^ ion. This suggests that both Zn^2+^ ions have a role in stabilizing the transition state for substrate hydrolysis. Overall, this study provides valuable insights into the challenges and considerations involved in studying the crystal structures of ligand–protein complexes, highlighting the importance of careful experimental design and rigorous data analysis in ensuring the accuracy and reliability of the resulting phosphotriesterase–organophosphate structures.

## Abstract 2

This study provides valuable insights into the challenges and considerations involved in the use of X-ray crystallography to study the 3D structures of ligand–protein complexes and highlights the importance of careful experimental design and rigorous data analysis in ensuring the validity of the structures obtained. A bacterial phosphotriesterase served as an experimental paradigm and novel insights were yielded into the role of the bimetal center of the enzyme in stabilizing the transition state for the hydrolysis of substrates.

## 1. Introduction

Structure-based drug design is a powerful tool in drug discovery, allowing researchers to predict how a potential drug will interact with its target protein. X-ray crystallography is widely used to obtain detailed structural information about the complex of a lead compound with the target protein, but also has certain limitations that may affect the accuracy and reliability of the structure determined. About 75% of the ∼175 000 macromolecular structures determined using X-ray crystallography in the Protein Data Bank (PDB; Berman *et al.*, 2000 ▸; Burley *et al.*, 2022 ▸; Sussman *et al.*, 1998 ▸) contain at least one of nearly 5000 unique ligands. Some ligands may be present serendipitously, due to the purification or crystallization processes (Dym *et al.*, 2016 ▸; Newman, 2004 ▸), while others are deliberately added to the sample to study protein function or in the context of structure-based drug design.

The experimental information that is utilized is the electron density of the protein and the ligand. The extent to which we can correctly interpret the electron density, and thus determine the positions of the atoms of both the protein and the ligand, determines the accuracy and reliability of the structure. Although stringent and strict validation tools are available for assessing the accuracy of a protein–ligand structure, in some of the structures deposited in the PDB overenthusiastic interpretation of the electron density of the ligand is encountered. This can lead to the placement of a ligand without supporting electron density. Another issue that may arise is incorrect assignment of the identity of the ligand (Dauter *et al.*, 2014 ▸; Weichenberger *et al.*, 2015 ▸). This can happen when the electron-density map is not clear enough to permit unambiguous interpretation. Finally, there may be cases in which the electron density cannot be fully accounted for, again leading to an uncertain or incorrect assignment. Thus, although crystallo­graphic studies of protein–ligand complexes provide a valuable experimental tool in drug discovery, they require careful interpretation and validation of the experimental data. Here, we discuss some of the difficulties encountered in studying organophosphate–phosphotriesterase complexes and how these difficulties were overcome.

**Figure 1:**
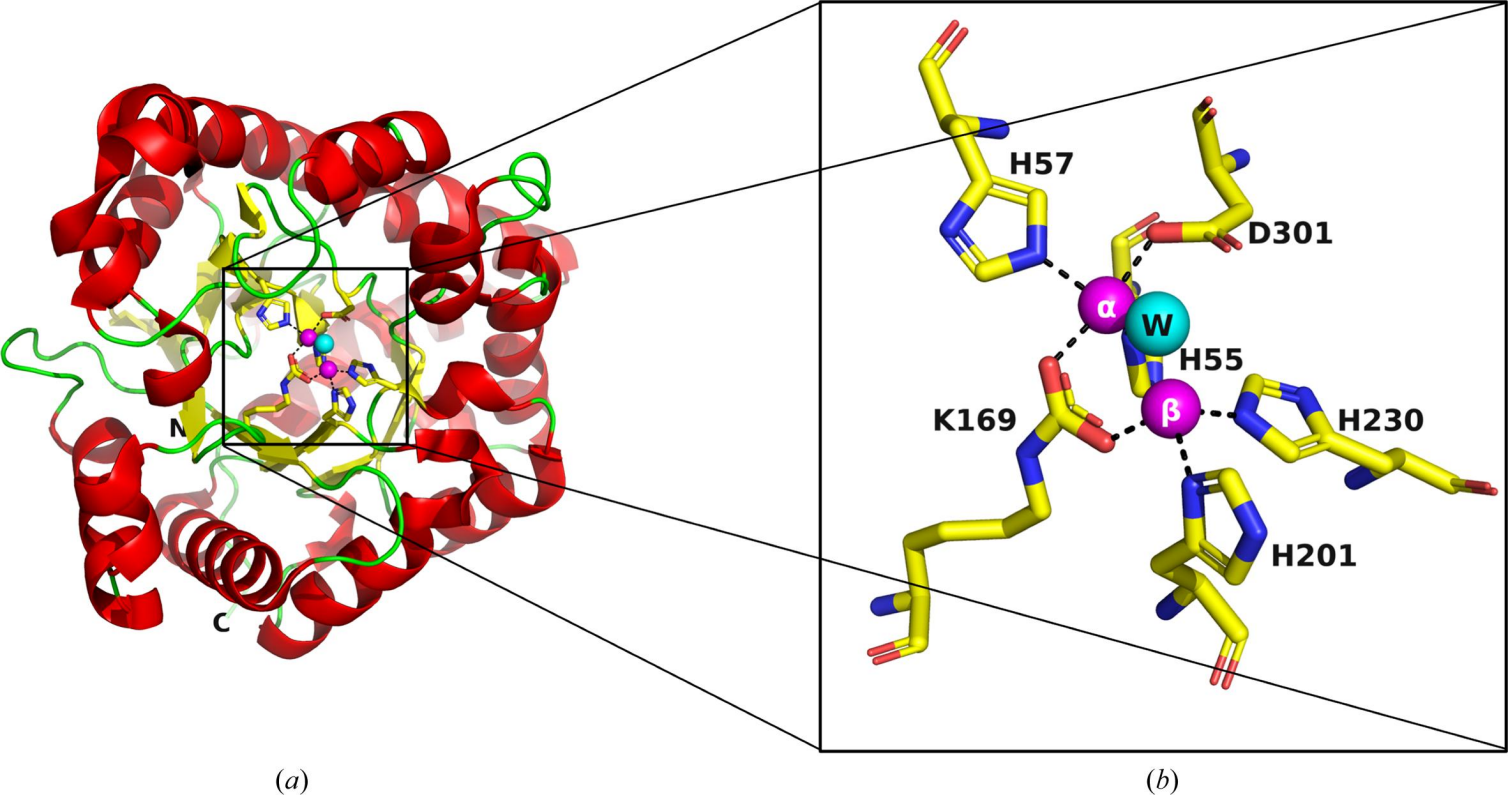
View of a typical PTE structure (PDB entry 1hzy; Benning *et al.*, 2001 ▸). (*a*) The (β/α)_8_ TIM-barrel fold is shown as a cartoon, with helices in red, sheets in yellow, coils in green, the α-Zn^2+^ (buried) and β-Zn^2+^ (exposed) ions as magenta spheres and a single bridging water shown as a cyan sphere. The six residues that bind to the two Zn^2+^ ions are shown as stick representations, with C atoms colored yellow, N atoms blue and O atoms red. The N- and C-terminal residues are labeled N and C, respectively. (*b*) Close-up view of the active site of the apo PTE structure. The buried α-Zn^2+^ ion is directly bound to His55, His57 and Asp301, while the exposed β-Zn^2+^ ion is bound to His201 and His230. The carbamate functional group bound to Lys169 interacts with both Zn^2+^ ions. Coloring is as in (*a*).

This study is based on our long-term interest in developing new therapies for treating organophosphate (OP) poisoning by insecticides and nerve agents. The enzyme phospho­triesterase (PTE) is capable of hydrolyzing such OP compounds. Accordingly, it has been characterized from a number of bacterial sources, including *Brevundimonas diminuta* (*Bd*; formerly *Pseudomonas diminuta*), *Flavobacterium* spp. and *Agrobacterium radiobacter* (Bigley *et al.*, 2013 ▸; Cherny *et al.*, 2013 ▸; Goldsmith *et al.*, 2016 ▸; Harper *et al.*, 1988 ▸; Horne *et al.*, 2002 ▸; Masson & Rochu, 2009 ▸). The present study focuses on *Bd*_PTE (Holm & Sander, 1997 ▸), a dimeric metallohydrolase that has a (β/α)_8_ TIM-barrel fold with two Zn^2+^ ions embedded in the active site (Fig. 1 ▸). Carbon dioxide reacts with the side chain of Lys169 to form a carbamate functional group within the active site, which serves as a bridging ligand to the α-Zn^2+^ and β-Zn^2+^ ions, both of which are required for full catalytic activity (Benning *et al.*, 1995 ▸). The buried α-Zn^2+^ ion is ligated to the protein via direct interactions with His55, His57 and Asp301, and the exposed β-Zn^2+^ ion interacts with His201 and His230 (Fig. 1 ▸ *b*).

There has been significant interest in utilizing enzymes that are capable of hydrolyzing OPs for the degradation and disposal of OP-based nerve agents and insecticides (Singh, 2009 ▸). Initial efforts to develop efficient enzyme variants for this purpose took human paraoxonase 1 (hPON1) as the starting point for multiple rounds of directed evolution, taken together with computational design and site-directed mutagenesis (Aharoni *et al.*, 2005 ▸; Gupta *et al.*, 2011 ▸; Worek *et al.*, 2014 ▸).

However, more recently, bacterial phosphotriesterases (PTEs) have proved to have a greater potential and have been used extensively over the course of the past two decades to generate variants with high catalytic efficiency towards a broad range of OPs of interest (Bigley *et al.*, 2015 ▸; Cherny *et al.*, 2013 ▸; Goldsmith *et al.*, 2016 ▸, 2017 ▸; Grimsley *et al.*, 2005 ▸; Jackson, Weir *et al.*, 2009 ▸; McLoughlin *et al.*, 2005 ▸; Tsai *et al.*, 2012 ▸; Yang *et al.*, 2003 ▸, 2014 ▸). Thus, variants have been produced that can serve as effective prophylactic and therapeutic antidotes for the treatment of both V-type and G-type nerve-agent intoxication (Cherny *et al.*, 2013 ▸; Despotović *et al.*, 2019 ▸; Goldsmith *et al.*, 2016 ▸, 2017 ▸). Recently, PTE isolated from a marine bacterium permitted growth on a pesticide analogue as the sole phosphorus source, suggesting that marine bacteria might serve for the remediation of diverse OP-containing pollutants (Despotović *et al.*, 2022 ▸).

**Figure 2:**
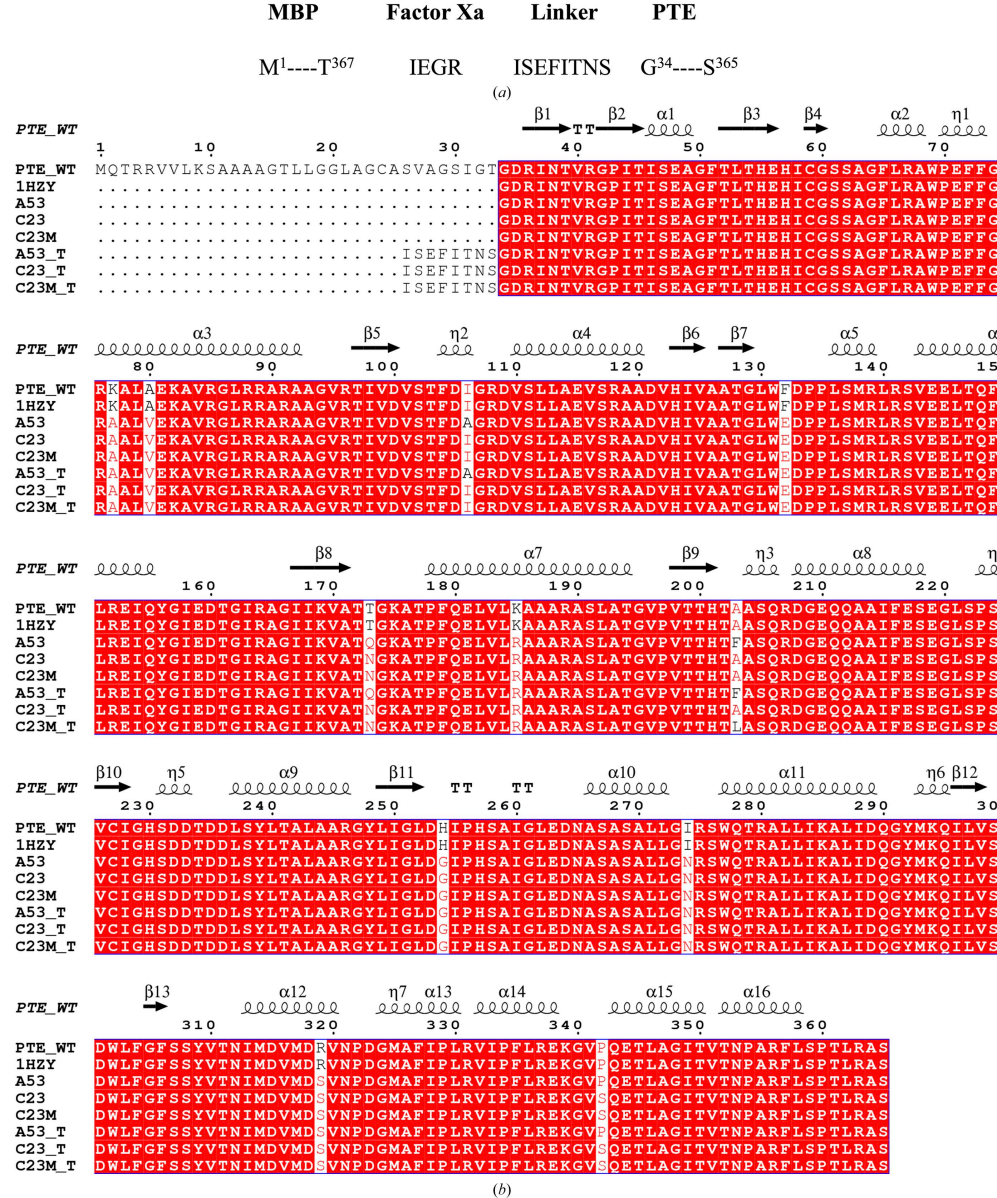
PTE variants. (*a*) Schematic presentation of the maltose-binding protein (MBP) fused before the factor Xa cleavage motif (IEGR) and the octapeptide spacer sequence, ^26^ISEFITNS^33^, followed by the mature PTE protein sequence starting with Gly34. Factor Xa cleaves after the arginine residue of the cleavage motif, leaving the ^26^ISEFITNS^33^ linker attached to the PTE, Gly34–Ser365. (*b*) Sequence alignment of wt PTE, PDB entry 1hzy, A53, C23, C23M, A53_T, C23_T and C23M_T. The last three bear the octapeptide tag. Secondary-structure elements of PDB entry 1hzy are labeled above the alignments: α-helices and 3_10_-helices (shown with the symbol η) are indicated by coils and β-strands by arrows. Residues conserved in all variants are in red. Multiple sequence alignment was performed using *MultAlin* (Corpet, 1988 ▸) and the figure was created using *ESPript* (Robert & Gouet, 2014 ▸).

Since the evolved PTE scaffolds had been shown to act as highly effective bioscavengers both *in vitro* and *in vivo*, it was important to clarify the structural features of their active sites. To date, 75 crystal structures of apo PTE and OP–PTE complexes, which are displayed in Table 1 ▸, have provided valuable information for mapping their active sites. We thought it important to determine the crystal structures of our advanced PTE variants and, when possible, those of their complexes with OPs so as to better understand the structural features that are responsible for their enhanced effectiveness. This study focused on three PTE variants, A53, C23 and C23M (Fig. 2 ▸ *b*), which correspond to G5-A53, G5-C23 and G5-B60, respectively, as described previously (Cherny *et al.*, 2013 ▸). These variants showed enhanced efficiency in degrading and detoxifying OPs relative to wild-type (wt) PTE. The following features were investigated.

(i) The effect of retaining a residual tag used for protein expression.

(ii) Three different crystallization conditions for A53, C23 and C23M were used to optimize the formation of PTE–OP complexes.

(iii) Four different space groups allowed the investigation of the impact of crystal packing on the formation of PTE–OP complexes.

(iv) The use of four different OP ligands permitted the identification of ligand-specific interactions and differences in these interactions between the three PTE variants.

## 2. Materials and methods

### 2.1. Expression and purification of PTE variants

PTE variants were expressed either without a fusion protein or tag, or as a fusion protein with maltose-binding protein (MBP). In the latter case, MBP was fused to PTE via a factor Xa cleavage motif sequence: IEGR (Cherny *et al.*, 2013 ▸) and a spacer sequence of eight amino-acid residues: ISEFITNS (see the schematic representations in Fig. 2 ▸ *a*). The purpose of using the MBP fusion partner was to increase the expression levels and solubility of the PTE variants. However, the MBP fusion partner was removed by digestion with factor Xa prior to crystallization trials.

#### 2.1.1. Expression of tag-free PTE variants

The PTE constructs A53, C23 and C23M (Cherny *et al.*, 2013 ▸) were generated based on a modified protocol (Tokuriki *et al.*, 2012 ▸; Fig. 2 ▸ *b*). These variants (residues Gly34–Ser365), devoid of any tag, were cloned into pET-21. They were grown in a 5 l culture of *Escherichia coli* BL21 cells at 30°C following induction with 0.5 m*M* isopropyl β-d-1-thiogalactopyranoside (IPTG). The bacterial pellet was resuspended in 20 m*M* HEPES pH 7.5 supplemented with 0.1 m*M* ZnCl_2_. The cells were sonicated for 2 min, employing pulses of 30 s on and 30 s off, at 35% amplitude in a Vibracell apparatus (Sonics & Materials Inc., Newtown, Connecticut, USA) at 4°C. The clarified lysate was loaded onto a HiTrap DEAE FF 5 ml column (GE Healthcare) pre-equilibrated with 0.1 m*M* ZnCl_2_, 20 m*M* HEPES pH 7.5. PTE-containing fractions were eluted with a 0–1 *M* NaCl gradient in the same buffer. The peak fractions containing PTE were dialyzed against 0.1 m*M* ZnCl_2_, 50 m*M* MES pH 6.0 and loaded onto a Tricorn Mono S 10/100 GL cation-exchange column (GE Healthcare) equilibrated with the same buffer. PTE was again eluted with a 0–1 *M* NaCl gradient in the same buffer. Fractions containing PTE were then concentrated to 5 ml and loaded onto a gel-filtration column (HiLoad Superdex 75 16/60) equilibrated with 50 m*M* NaCl, 25 m*M* HEPES pH 8.0. The pooled eluent fractions were applied onto a Tricorn Q 10/100 GL anion-exchange column equilibrated with 50 m*M* Tris pH 8.0 and eluted with a 0–1 *M* NaCl gradient in the same buffer. The final protein solution was concentrated to 13–17 mg ml^−1^ for crystallization screening.

#### 2.1.2. Expression and purification of MBP-PTE fusion proteins

To increase the expression levels and solubility of PTE, maltose-binding protein (MBP) was introduced into the three PTE variants as a fusion partner, together with a factor Xa cleavage motif squence: IEGR (Zhao *et al.*, 2013 ▸), followed by an octapeptide spacer (ISEFITNS) preceding PTE (Fig. 2 ▸ *a*). These fusion proteins were expressed and purified as described previously (Cherny *et al.*, 2013 ▸). Briefly, *E. coli* BL21 cells were transformed with a PTE plasmid bearing the desired mutation. The *E. coli* BL21 cells were grown overnight at 37°C in lysogeny broth (LB; Bertani, 1951 ▸) supplemented with ampicillin (100 µg ml^−1^). They were then subcultured [1%(*v*/*v*) inoculum] into LB supplemented with 0.2 m*M* ZnCl_2_ and ampicillin (100 µg ml^−1^) and allowed to grow at 37°C until OD_600 nm_ reached 0.6–0.8, followed by induction with 0.4 m*M* IPTG and further growth for 14–18 h at 20°C. The cell pellet was suspended in buffer *A* (0.1 m*M* ZnCl_2_, 10 m*M* NaHCO_3_, 100 m*M* NaCl, 100 m*M* Tris pH 8.0) containing 0.4 mg ml^−1^ lysozyme, 50 units of Benzonase and 1 m*M* phenylmethyl­sulfonyl fluoride, and sonicated as described for the unpacked protein. A clear cell lysate was obtained by centrifugation at 7500 rev min^−1^ for 30 min. Amylose beads equilibrated with buffer *A* were packed into a 10 ml column. The clarified lysate was loaded onto the column by gravitation and washed with buffer *A*. The MBP-PTE fusion variants were eluted with 10 m*M* maltose in buffer *A*. The eluted fractions were analyzed by 12% SDS–PAGE. Fractions containing MBP-PTE were pooled and dialyzed overnight against buffer *A*. The protein concentration was measured at 280 nm using a molar extinction coefficient for MBP-PTE of 95 800 cm^−1^ *M* ^−1^.

For crystallization trials, the A53, C23 and C23M variants were expressed and purified from 2.5–5 l cultures as above. The suspended pellet was disrupted using a cell disruptor (Constant Systems, Low March, United Kingdom), clarified by centrifugation and applied onto a column containing amylose beads. MBP-PTE variants were eluted with maltose, and MBP was cleaved by incubation with 25 µg factor Xa per 800 ml culture (Zhao *et al.*, 2013 ▸) for 40 h at 4°C. Post-cleavage, the solution was passed four times over a column packed with fresh amylose beads to retain the cleaved MBP and any residual uncleaved MBP-PTE, while tagless PTE appeared in the flowthrough. In some cases where PTE was still contaminated with residual MBP, an anion-exchange column was used to separate the tagless PTE from residual MBP. In these cases, the protein solution was dialyzed against 0.1 m*M* ZnCl_2_, 10% glycerol, 200 m*M* NaCl, 20 m*M* Tris pH 8.0 and loaded onto a Tricorn Q10/100 column (GE Healthcare). Under these conditions, pure PTE was obtained in the flowthrough of the column, while MBP was retained. The tagless PTE was dialyzed against buffer *A* and concentrated to ∼11 mg ml^−1^.

### 2.2. Synthesis of OP ligands

#### 2.2.1. Methylphosphonic acid

**Figure 3:**
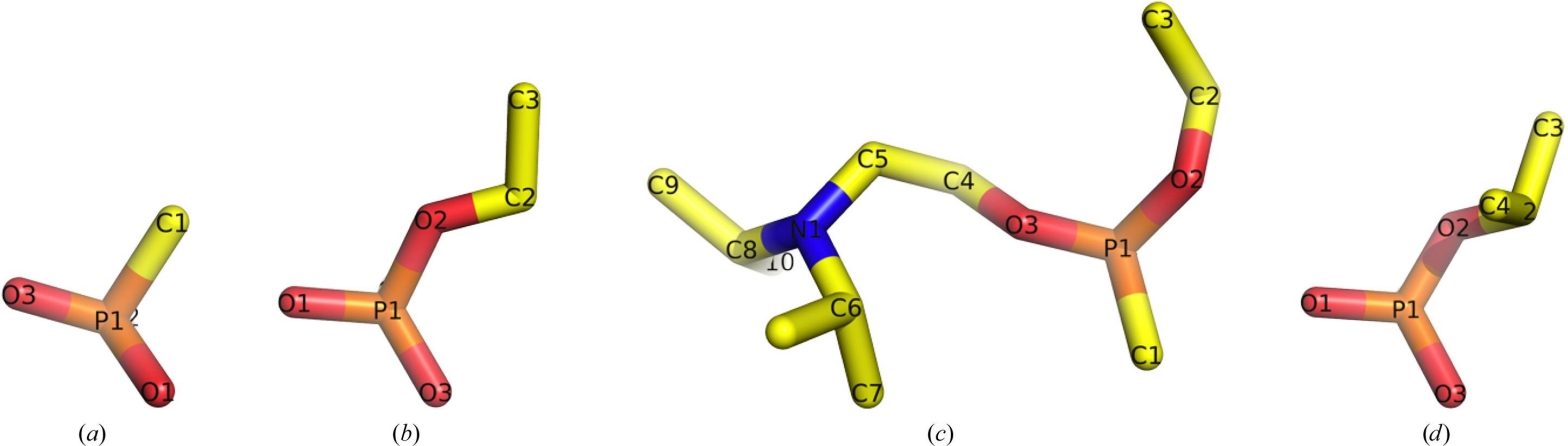
Methylphosphonates that were crystallized with the PTE variants. (*a*) Methylphosphonic acid, (*b*) *O*-ethyl methylphosphonic acid, (*c*) *O*-ethyl-*O*-(*N*,*N*-diisopropylaminoethyl) methylphosphonate (the oxo analogue of VX), (*d*) *O*-isopropyl methylphosphonic acid. In all four structures the CH_3_ moiety is pointing away from the viewing plane and thus is not seen. The OPs are shown as stick figures with C atoms colored yellow, N atoms blue, O atoms red and P atoms orange.

Methylphosphonic acid (Fig. 3 ▸ *a*) was obtained by the dropwise addition of 266 mg methylphosphonyl dichloride in 3 ml acetone to 5 ml H_2_O at 4°C. After three days at room temperature (RT) the mixture of water and acetone was removed under vacuum to yield a viscous oil, which then solidified. Only one kind of ^31^P-NMR signal was observed, with a chemical shift (δ) of 34.0 p.p.m. in D_2_O. This signal corresponds to the resonance of the P atom in methylphosphonic acid (Fig. 3 ▸ *a*).

#### 2.2.2. *O*-Ethyl methylphosphonic acid

A solution of 100 mg *O*-ethyl methylphosphonyl chloride (Levy & Ashani, 1986 ▸) in 5 ml acetone was added dropwise to 10 ml H_2_O at 4°C and stirred for 30 min. The mixture of water and acetone was removed under vacuum to yield a viscous oil. The ^31^P-NMR δ was 35.5 p.p.m. in D_2_O. This signal corresponds to the resonance of the P atom in *O*-ethyl methylphosphonic acid (Fig. 3 ▸ *b*).

#### 2.2.3. *O*-Ethyl-*O*-(*N*,*N*-diisopropylaminoethyl) methylphos­phonate

*O*-Ethyl methylphosphonyl chloride (7.7 g in 25 ml dry ether) was added dropwise to a cold (ice-bath) solution of equimolar amounts of 5 g triethylamine and 7.5 g *N*,*N*-diisopropylaminoethanol in 30 ml dry ether to obtain *O*-ethyl-*O*-(*N*,*N*-diisopropylaminoethyl) methylphosphonate (Fig. 3 ▸ *c*). After stirring for 48 h at RT, the precipitated solid was filtered off and the ether was washed several times with cold distilled water. The ether was dried over anhydrous sodium sulfate, filtered and removed under vacuum to leave behind 8.5 g of a pale yellow viscous oil. A ^31^P-NMR signal was observed at a δ of 33.8 p.p.m. in CDCl_3_. This signal corresponds to the resonance of the P atom in *O*-ethyl-*O*-(*N*,*N*-diisopropylamino­ethyl) methylphosphonate (Fig. 3 ▸ *c*). The chemical shifts and signal patterns of the ^1^H-NMR spectrum (300 MHz, CDCl_3_) are consistent with the structure of the purified ligand. δ: 4.08 (m, 10 lines, 2H), 2.85 (m, 10 lines, 2H), 2.97 (septate, 2H), 2.65 (triplet, 3H), 1.45 (doublet, 3H), 1.30 (triplet, 3H), 0.96 (doublet, 12H).

#### 2.2.4. *O*-Isopropyl methylphosphonic acid

*O*-Isopropyl methylphosphonyl chloride was synthesized by reacting *O*,*O*-diisopropyl methylphosphonate with oxalyl chloride following the method described above for *O*-ethyl methylphosphonyl chloride. Thus, 7.8 g *O*-isopropyl methylphos­phonyl chloride was added dropwise, while stirring, to 60 ml water precooled in an ice bath. After 60 min, water was removed under vacuum and the residual oil was dissolved in 100 ml benzene. The organic solution was dried over anhydrous sodium sulfate, filtered and evaporated under vacuum, leaving behind ∼5 g of a colorless oil. Only one kind of ^31^P-NMR signal was observed, with a δ of 36.0 p.p.m. in D_2_O. This signal corresponds to the resonance of the P atom in *O*-isopropyl methylphosphonic acid (Fig. 3 ▸ *d*).

#### 2.2.5. Determination of the dissociation constants of the complexes between wt PTE and OP product ligands

In order to determine the association strength between PTE and the two acid products, methylphosphonic acid (Fig. 3 ▸ *a*) and *O*-ethyl methylphosphonic acid (Fig. 3 ▸ *b*), the dissociation constants of the corresponding complexes were evaluated by use of the secondary plots of Lineweaver–Burk slopes (Radić *et al.*, 1993 ▸). Increasing amounts of the OP acid were added to 1 m*M* paraoxon in 50 m*M* NaCl, 50 m*M* Tris pH 8.0 containing 0.1% Tergitol. The initial velocity of hydrolysis of paraoxon by wt PTE was recorded over the first 2 min by monitoring the release of *p*-nitrophenol at 400 nm.

### 2.3. Crystallization and structure determination of PTE variants

Crystals of the three PTE variants, A53, C23 and C23M, were grown at 19°C by the hanging-drop vapor-diffusion technique using a Mosquito robot (SPT Labtech, Cambridge, Massachusetts, USA). Crystals of the PTE variants were obtained under three different crystallization conditions: (i) polyethylene glycol (PEG) 6000 and 2-methyl-2,4-pentanediol (MPD), (ii) glycerol and ammonium sulfate (AS) and (iii) polyacrylic acid (PAA). The crystals grew in four different space groups: *P*2_1_2_1_2 (PEG 6000 and MPD, A53_1), *P*2_1_2_1_2_1_ (PEG 6000 and MPD, C23_1), *P*2_1_ (PEG 6000 and MPD, A53_3; PAA, A53_4) and *P*4_3_2_1_2 (AS and glycerol, A53_2, A53_5, C23_2, C23_3, C23_4, C23_5, C23M_1 and C23M_2). With one exception, C23_1, the crystals diffracted to high resolutions in the range 1.38–2.00 Å. Crystallization optim­ization experiments were set up manually using the hanging-drop vapor-diffusion technique. Four methylphosphonate ligands were co-crystallized with the A53, C23 and C23M variants (Fig. 3 ▸). 12 crystal structures were analyzed, including the apo A53, C23 and C23M variants, as well as several complexes with the phosphonate ligands.

X-ray data collection was performed under cryo-conditions (100 K) either in-house on a Rigaku RU-H3R instrument or on beamlines ID14-4 and ID23-1 at the ESRF, Grenoble, France (Table 2 ▸). All diffraction images were indexed and integrated using *MOSFLM* (Leslie & Powell, 2007 ▸) and the integrated reflections were scaled using *SCALA* (Evans, 2006 ▸). Structure-factor amplitudes were calculated using *TRUNCATE* (French & Wilson, 1978 ▸) from the *CCP*4 suite (Agirre *et al.*, 2023 ▸). The structure of apo A53 (A53_1) was solved by molecular replacement with *Phaser* (McCoy *et al.*, 2007 ▸) using the homologous refined structure of *Bd*_PTE (PDB entry 1hzy; Benning *et al.*, 2001 ▸) as a starting model. All steps of atomic refinement were performed with *REFMAC* (Murshudov *et al.*, 2011 ▸) and *Phenix* (Liebschner *et al.*, 2019 ▸). The models were built into the electron-density map using *Coot* (Emsley *et al.*, 2010 ▸). Following this, the refined A53_1 structure was used as a model to determine the structures of all of the other PTE variants (Table 2 ▸). All models were optimized using *PDB-REDO* (Joosten *et al.*, 2014 ▸) and were evaluated with *MolProbity* (Chen *et al.*, 2010 ▸). Details of the refinement statistics are presented in Table 2 ▸. The coordinates of the A53_1, A53_2, A53_3, A53_4, A53_5, C23_1, C23_2, C23_3, C23_4, C23_5, C23M_1 and C23M_2 structures have been deposited in the Protein Data Bank under PDB codes 8p7f, 8p7h, 8p7i, 8p7k, 8p7m, 8p7n, 8p7q, 8p7r, 8p7s, 8p7t, 8p7u and 8p7v, respectively.

## 3. Results

### 3.1. Correlation between the presence of Zn^2+^ and the absence of tags: A53_1 and C23_1

The A53 variant, which is devoid of any tags, was crystallized in the presence of 12% PEG 6000, 5% MPD, 0.1 *M* HEPES pH 7.5 and the crystals diffracted to 2.0 Å resolution (A53_1 in Table 2 ▸). The (β/α)_8_ TIM-barrel fold of A53_1 is very similar to that of wt *Bd*_PTE (PDB entry 1hzy; Benning *et al.*, 2001 ▸). However, A53_1 is a monomer, with only one Zn^2+^ ion in the active site. PTEs require two divalent metal ions in their active site, which contribute to both their activity and their stability (Benning *et al.*, 1995 ▸). The removal of either of the two metal ions results in a loss of enzymatic activity. The exposed α-Zn^2+^ ion in the PTE structures, as seen, for example, in PDB entry 1hzy (Benning *et al.*, 2001 ▸), is ligated by His55, His57 and Asp301, and the buried β-Zn^2+^ ion is ligated by His201 and His230, while the carbamate functional group bound to Lys169 interacts with both Zn^2+^ ions (Fig. 1 ▸ *b*). These findings are consistent with the general observation in metalloenzymes that a hydrophilic active site is embedded in a hydrophobic core (Yamashita *et al.*, 1990 ▸).

**Figure 4:**
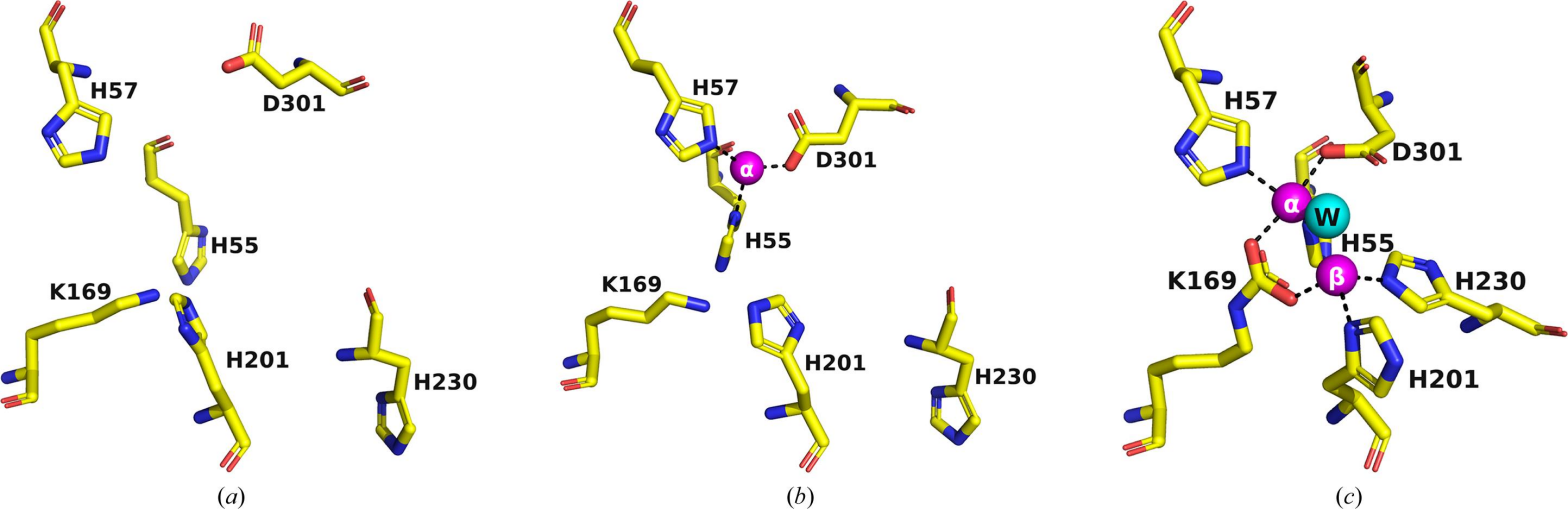
Comparison of the active-site regions of PTEs containing different numbers of Zn^2+^ ions. (*a*) PDB entry 1pta, which is devoid of Zn^2+^ ions. (*b*) A53_1, containing one Zn^2+^ ion. (*c*) PDB entry 1hzy, containing two Zn^2+^ ions. Zn^2+^ ions are shown in magenta and the bridging water is in cyan.

A53_1 and PDB entry 1pta (Benning *et al.*, 1994 ▸) crystallize in the same space group *P*2_1_2_1_2, with virtually identical unit cells: *a* = 79.7, *b* = 93.7, *c* = 44.6 Å and *a* = 80.2, *b* = 93.7, *c* = 45.0 Å, respectively. It is interesting that the structure with PDB code 1pta does not contain any Zn^2+^ ions in its active site (Fig. 4 ▸ *a*), despite being structurally very similar to the A53_1 variant (r.m.s.d. of 0.498 Å), which contains one Zn^2+^ ion, corresponding to the buried α-Zn^2+^ ion, in its active site (Fig. 4 ▸ *b*). The putative active sites of both the PDB entry 1pta and A53_1 structures differ significantly from the canonical active site containing two Zn^2+^ ions, as is the case for other PTE structures, such as PDB entry 1hzy (Fig. 4 ▸ *c*).

It has been reported that some reagents used in crystallization processes can act as chelators, creating coordinate bonds to the Zn^2+^ ions in solution and thus decreasing their effective concentration, with Tris being one such reagent (Fischer *et al.*, 1979 ▸). In the present study, Tris was used at high concentrations, >50 m*M*, both in the purification of the PTE variants and in some of the crystallization trials. Tris can chelate metal ions, especially Zn^2+^, via its amine N atom (Handing *et al.*, 2018 ▸). Its presence in the purification buffer thus decreases the effective concentration of free Zn^2+^, making it difficult to incorporate two Zn^2+^ ions into the active sites of the PTE variants. Since the presence of both Zn^2+^ ions and the correct orientation of the residues in the active site are crucial for maintaining the catalytic activity of PTE, it is reasonable to assume that the A53_1 monomer is catalytically inactive. To avoid forming inactive PTE molecules containing less than two Zn^2+^ ions in the active site, it was crucial to add ZnCl_2_ during protein expression, purification and crystallization. All of the structures listed in Table 2 ▸, except for A53_1 and C23_1, were obtained using preparations in which the purification media were supplemented with ZnCl_2_, and indeed display molecular dimers with two Zn^2+^ ions in the active site of each monomer.

Comparison of PDB entry 1hzy, containing two Zn^2+^ ions, with A53_1, which contains only one, reveals an r.m.s.d. of 0.65 Å on C^α^ atoms. Inspection of the active site of A53_1 shows that the α-Zn^2+^ ion is ∼2.5 Å away from the corresponding ion in PDB entry 1hzy (Figs. 4 ▸ *b* and 4 ▸ *c*, respectively). Moreover, there are noticeable conformational deviations in key active-site residues (Fig. 4 ▸). These deviations can be attributed to the absence of the β-Zn^2+^ ion, which would have been expected to coordinate to the side chains of His201 and His230 (Figs. 1 ▸ *b* and 4 ▸ *c*). Interestingly, the structure of A53_1 more closely resembles that of PDB entry 1pta, which lacks any Zn^2+^ ions (Figs. 4 ▸ *b* and 4 ▸ *a*, respectively).

**Figure 5:**
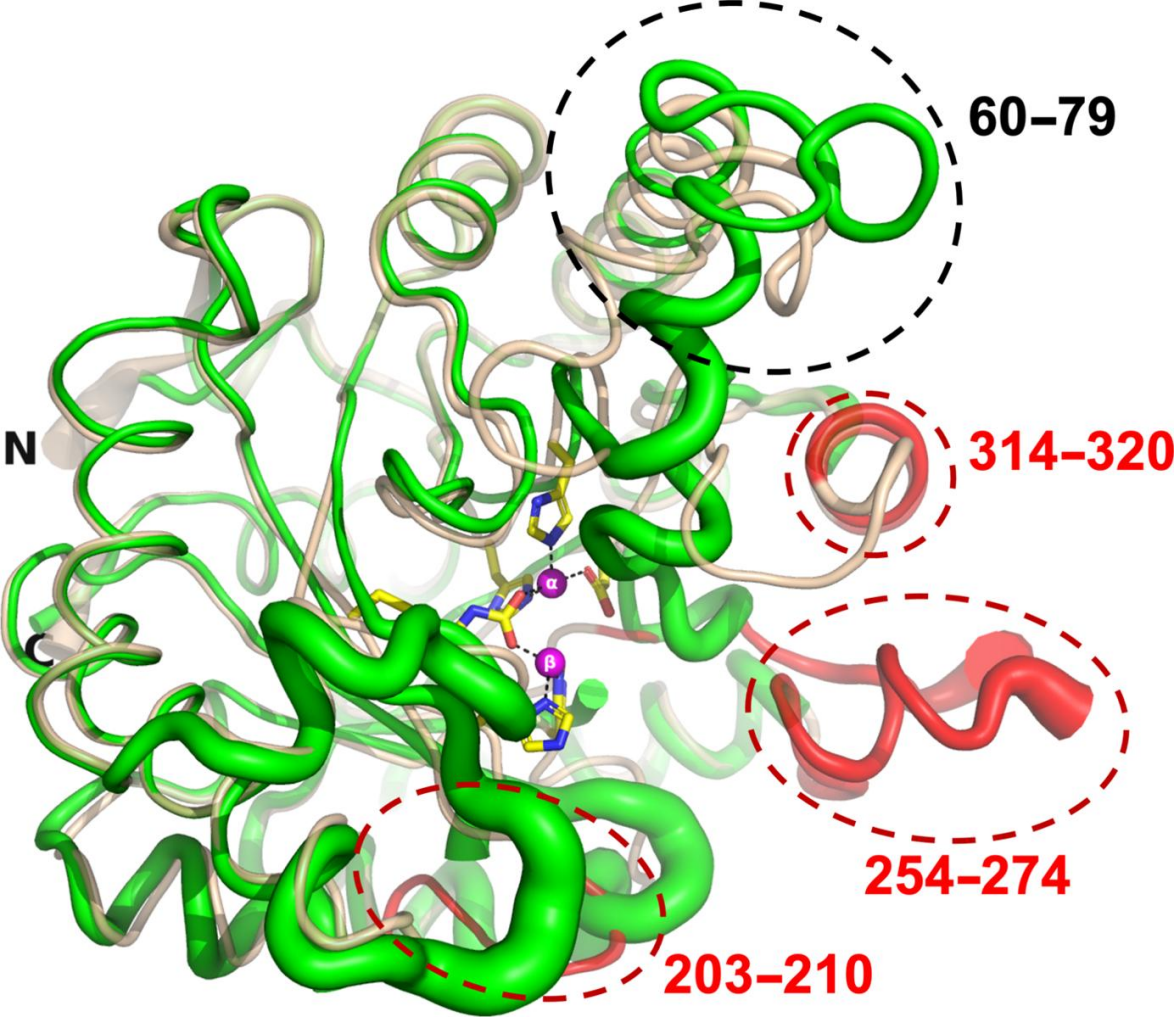
Cartoon tube diagrams of the backbones of the monomer structures of apo A53_2 (beige) and apo A53_1 (green). A region displaying sizeable conformational differences (residues 60–79) is circled by a black dashed line. This region participates in dimer formation in the A53_2, A53_3 and A53_4 structures, which are very similar and differ significantly from that of A53_1. The missing regions in A53_1, *i.e.* residues 203–210, 254–274 and 314–320, are colored red and circled by red dashed lines. The two Zn^2+^ ions in A53_2 are shown as magenta spheres and the residues which bind them are displayed as sticks.

While almost all of the PTEs in the PDB are seen to crystallize with molecular dimers in the asymmetric unit, A53_1 displays only one molecule in the asymmetric unit. A dimer is formed by applying the crystallographic twofold axis in space group *P*2_1_2_1_2. However, this dimer interface utilizes different residues and is rather loose compared with the canonical non­crystallographic dimer seen in other PTEs, suggesting that it is not a physiological dimer. The two segments, residues 60–79 and 301–313, which are involved in the canonical PTE dimer interface show pronounced conformational differences in the A53_1 structure cartoon shown in Fig. 5 ▸. Furthermore, three regions, 203–209, 254–275 and 314–320, are disordered and thus are not visible in the electron-density map (Fig. 5 ▸). Residues 314–320 are close to residues 301–313, which are involved in the canonical PTE dimer interface. Since some of the residues in the disordered regions are in the vicinity of the active site, it is plausible that their disorder, and the significant conformational changes observed in residues 60–79 and 301–313, as well as the presence of only one Zn^2+^ ion, preclude the dimerization of the A53_1 structure and eliminate catalytic activity. Indeed, A53_1 occurs as a monomer in solution, as demonstrated by gel filtration (not shown).

The tag-free C23 variant was expressed without the MBP fusion tag and purified by conventional purification tech­niques. Crystals of C23_1 obtained from 10% PEG 6000, 15% MPD, 2% PEG 400, 0.1 *M* HEPES pH 7.5 diffracted to 3.2 Å resolution (Table 2 ▸). Since the crystals diffracted so poorly, it was deemed to be unsuitable for use in OP co-crystallization experiments.

### 3.2. The structures of tagged constructs of A53, C23 and C23M

The addition of ZnCl_2_ during expression, purification and crystallization resulted in the presence of two Zn^2+^ ions in the active sites of A53_2, A53_3 and A53_4, all three of which crystallized under different crystallization conditions. The coordination geometry of the two Zn^2+^ ions in the active site of these three structures is similar to that observed in other PTE structures, such as PDB entry 1hzy. Interestingly, the crystal structure of A53_2 has a monomer in the asymmetric unit and the dimer is formed through a crystallographic twofold axis in space group *P*4_3_2_1_2, as observed in the structures of several other PTEs: A53_5, C23_2, C23_3, C23_4, C23_5, C23M_1 and C23M_2. However, A53_3 and A53_4 crystallize in space group *P*2_1_ with a dimer in the asymmetric unit (Table 2 ▸).

### 3.3. Impact of a residual tag on the apo structures of the A53, C23 and C23M variants

To facilitate purification, the A53, C23 and C23M variants were expressed as constructs with an N-terminal MBP tag (Fig. 2 ▸ *a*). Factor Xa cleavage resulted in proteins with an octapeptide linker (ISEFITNS) at the N-terminus followed by the mature PTE protein sequence, starting at Gly34, for constructs A53_T, C23_T and C23M_T (Fig. 2 ▸ *b*).

Apo structures of the octapeptide-tagged A53 variant were obtained using three different crystallization precipitants: AS with glycerol, PEG 6000 with MPD, and PAA, which resulted in crystal structures A53_2, A53_3 and A53_4, respectively. Crystals of apo C23 were obtained using three precipitants: PEG 6000 with MPD (C23_1), PAA, and AS with glycerol (data not shown).

C23_1 crystallized in space group *P*2_1_2_1_2_1_, with two dimers in the asymmetric unit. The crystals of tagged C23 (C23_2, C23_3, C23_4 and C23_5) and C23M (C23M_1 and C23M_2) grew from AS and glycerol, with one monomer in space group P4_3_2_1_2, such that the canonical dimer is generated by crystallographic symmetry.

**Figure 6:**
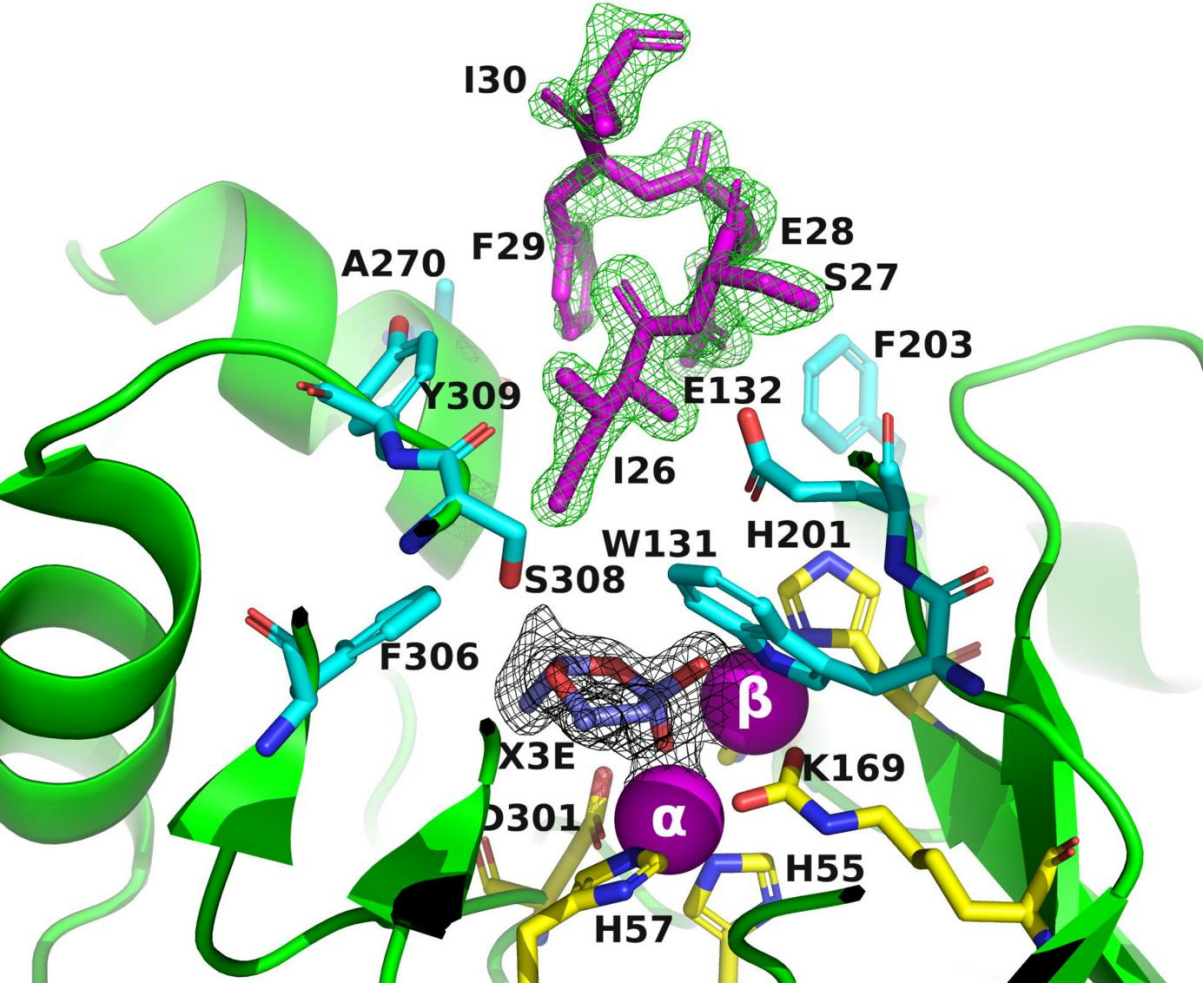
Ribbon representation of the A53_3 variant. It shows the octapeptide tag on subunit *B* penetrating the active-site region of the symmetry-related subunit *A*. The tag is shown in magenta. The active-site residues of the symmetrically related chain *A* are shown in yellow, with those residues within 5 Å of the tag shown in cyan. The green electron density corresponds to an omit map with the octapeptide omitted (contoured at 3σ). The cyclic compound X3E, which was presumably carried over from the protein expression and purification process, is seen in the active site, and the black electron density corresponds to a 2*F*_o_ − *F*_c_ map (contoured at 1σ). The two Zn^2+^ ions are shown as magenta spheres.

In the crystal structures of A53_3 and A53_4, electron density corresponding to residues from the octapeptide spacer ^26^ISEFITNS^33^ was unexpectedly observed (A53_T in Fig. 2 ▸ *b*). Thus, the residual octapeptide of subunit *B* was found to penetrate into the active site of the symmetry-related subunit *A*, such that in A53_3 the distance between the active site of the *A* subunit and the N-terminus (Ile26) of the octapeptide of subunit *B* was approximately 4.5 Å (Fig. 6 ▸). The residues from the octapeptide of subunit *B* made contact with Trp131, Glu132, Gln173, Phe203, Ala270, Phe306, Ser308 and Tyr309 in the symmetry-related subunit *A*. Interestingly, although the crystals of A53_3 and A53_4 were obtained from different crystallization conditions (PEG 6000 with MPD and PAA, respectively), both crystallized in space group *P*2_1_ and both retained the residual octapeptide. Notably, A53 crystallized in the presence of PEG 8000 in space group *P*2_1_ also contains the residual octapeptide in the active site (data not shown). Since the octapeptide was only seen in crystals in space group *P*2_1_, it is reasonable to assume that penetration of the residual octapeptide into the active site is space-group dependent. The presence of the octapeptide in the active site might be expected to interfere with the binding of OPs. This indeed explains why no OPs were found in crystals of A53_3 and A53_4 (Fig. 6 ▸). The octapeptide behaves like a peptide-inhibitor mimic and thus may help to define the mode of binding of a substrate with a long side chain.

### 3.4. PTE crystal structures obtained using polyacrylic acid as the precipitant

**Figure 7:**
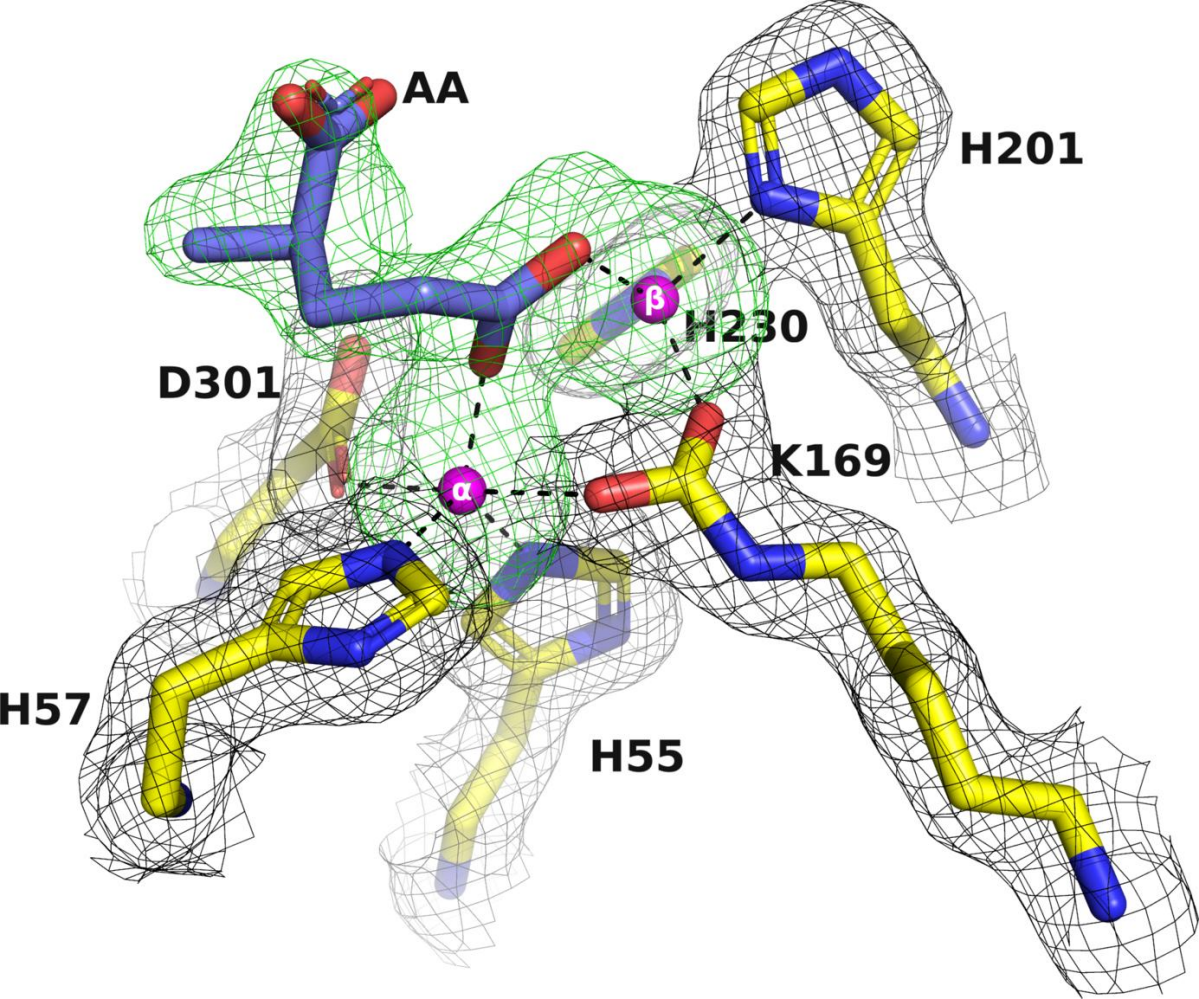
Active-site region of A53_4. Acrylic acid (AA; blue sticks) is clearly seen bound at the active site. The green electron density corresponds to an omit map with AA and the two Zn^2+^ ions omitted (contoured at 3σ). The black electron density corresponds to a 2*F*_o_ − *F*_c_ map (contoured at 1σ). The two Zn^2+^ ions are shown as magenta spheres.

PTE variants were also crystallized using polyacrylic acid (PAA), including A53 (A53_4; Table 2 ▸) and C23 (data not shown). In these cases, no water molecules are directly bound to the Zn^2+^ ions, since the PAA monomer, *i.e.* acrylic acid (AA), is detected in the active site. The bound AA acts as a ligand to bridge the two Zn^2+^ metal ions, mimicking the tetrahedral intermediate formed during the hydrolysis of carboxylate esters and OPs (Fig. 7 ▸). The presence of crystallization reagents in the active site of PTE is not unusual, since similar observations have also been made in other PTE structures (Table 2 ▸). Thus, cacodylate was observed in many PTE structures, with its two O atoms bound to the two Zn^2+^ ions (for example, PDB entries 4xd3, 4xd4, 4xd5, 4xd6, 4xaf, 4xag, 4xay, 6gbl and others; Campbell *et al.*, 2016 ▸). In the structure of organophosphorus acid anhydrolase (OPAA; PDB entry 4zwo), glycolic acid, which resembles acrylic acid, was observed in the active site, with its two O atoms bound to the two Mn^2+^ ions (Daczkowski *et al.*, 2015 ▸). Many studies have been published on nasal drug delivery making use of PAA derivatives, and its bioadhesive properties are well recognized (Arkaban *et al.*, 2022 ▸; Sabale *et al.*, 2020 ▸). Indeed, its strong bioadhesive properties and its high capacity to bind to proteins (Dai *et al.*, 2006 ▸) can explain its presence within the active site of the PTE variants. Furthermore, in addition to AA, the A53_4 active site also contains the octapeptide, which is exclusively observed in space group *P*2_1_ (as described in Section 3.3). This suggests that the crystallization reagent PAA initially binds to the active site of PTE in solution. Subsequently, the octapeptide spacer ^26^ISEFITNS^33^ penetrates the active site during crystal formation in space group *P*2_1_. Since no electron density for the co-crystallized OP ligands was observed in any PTE variants crystallized from PAA, the latter may have a higher affinity for the Zn^2+^ ions than the OPs.

### 3.5. Identifying the metal ion within the active site of PTE

**Figure 8:**
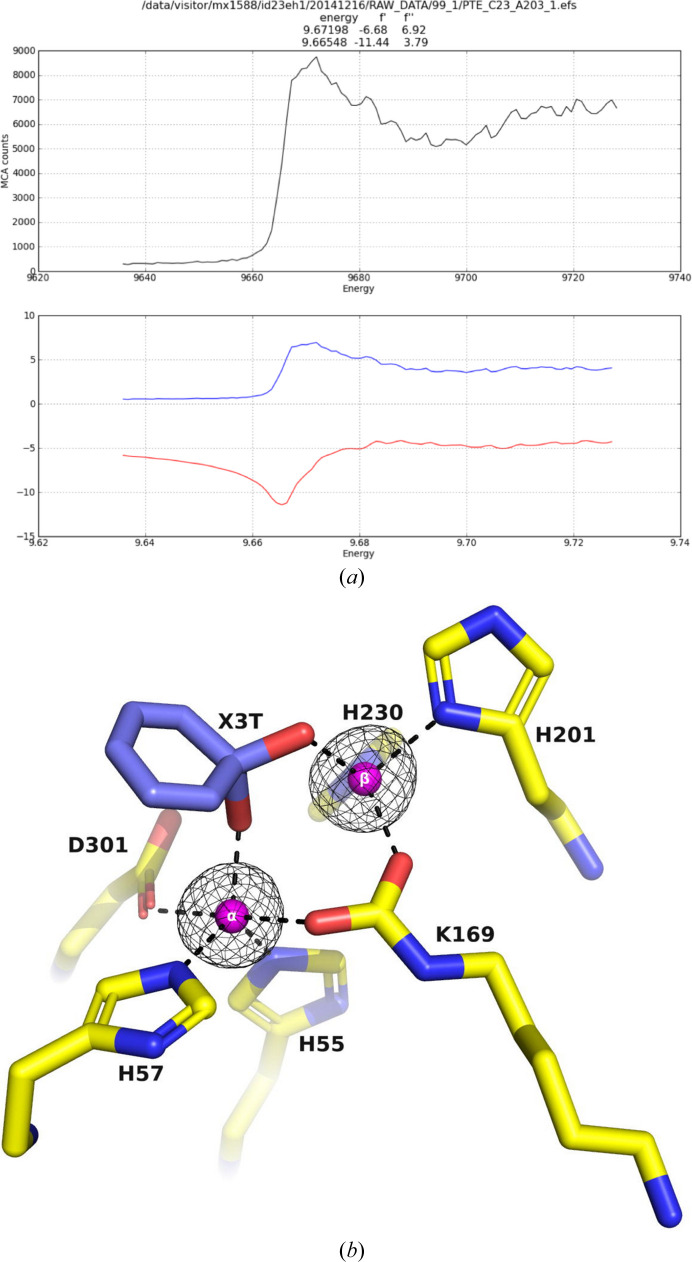
(*a*) Scan of the C23M_1 crystal at a range of energies showing a peak corresponding to the zinc absorption edge (top). Scattering factors (*f*′ and *f*′′) are plotted as a function of energy (bottom). (*b*) An anomalous omit electron-density map of the active-site region of C23M_1, contoured at 6σ, is shown in black. The two Zn^2+^ ions are shown as magenta spheres and were omitted in calculating the electron density.

Identifying the intrinsically bound metal ion(s) in a protein–metal complex structure is crucial to ensure that they are consistent with the solutions employed in the expression, purification and crystallization steps (Zheng *et al.*, 2008 ▸). Unanticipated metal ions can potentially replace the expected ones, resulting in incorrect or misleading data. In the case of the PTE variants, it was essential to confirm the identity of the metal ions in the active site. Accordingly, the X-ray data for C23M_1 crystallized from AS and glycerol were collected at the zinc absorption-edge wavelength (λ = 1.2724 Å) on beamline ID23-1 at the ESRF from a crystal that diffracted to 1.38 Å resolution (C23M_1 in Table 2 ▸; Fig. 8 ▸ *a*), showing a peak corresponding to the zinc absorption edge. The anomalous omit electron-density maps of the active-site region show unequivocally that the two metal ions within the active site are indeed both Zn^2+^ ions, thus confirming their expected identity (Figs. 8 ▸ *b* and 9 ▸).

It can be seen that there is well defined electron density in the active site of C23M_1 corresponding to an unidentified six-membered ring ligand (X3T). This ligand will be discussed in more detail in the following section.

### 3.6. Cyclic compounds in PTE active sites

As already noted, it is not uncommon for the crystallization precipitants and compounds used in protein expression and purification to be observed within the active sites of protein structures (Dym *et al.*, 2016 ▸). PEG and MPD were used as precipitants for the crystallization of A53_1, A53_3 and C23_1 (Table 2 ▸). As described above, A53_1 is presumably an inactive monomer with one Zn^2+^ ion in its active site and C23_1 diffracted to low resolution. A53_3 crystallized in space group *P*2_1_, with the residual octapeptide pointing into the active site. Additionally, an unidentified six-membered ring ligand (X3E) was observed in the active site with two O atoms, likely acting as a chelator that coordinates the two Zn^2+^ ions present in the active site of PTE (Fig. 6 ▸). For A53, which was crystallized from PEG 6000 and MPD, no electron density for the co-crystallized OP ligands was detected, as was the case for A53 crystallized from PAA. Interestingly, both A53_3 and A53_4, which contain X3E and AA, respectively, in their active sites, crystallized in space group *P*2_1_, with the residual octapeptide pointing into their active site.

**Figure 9:**
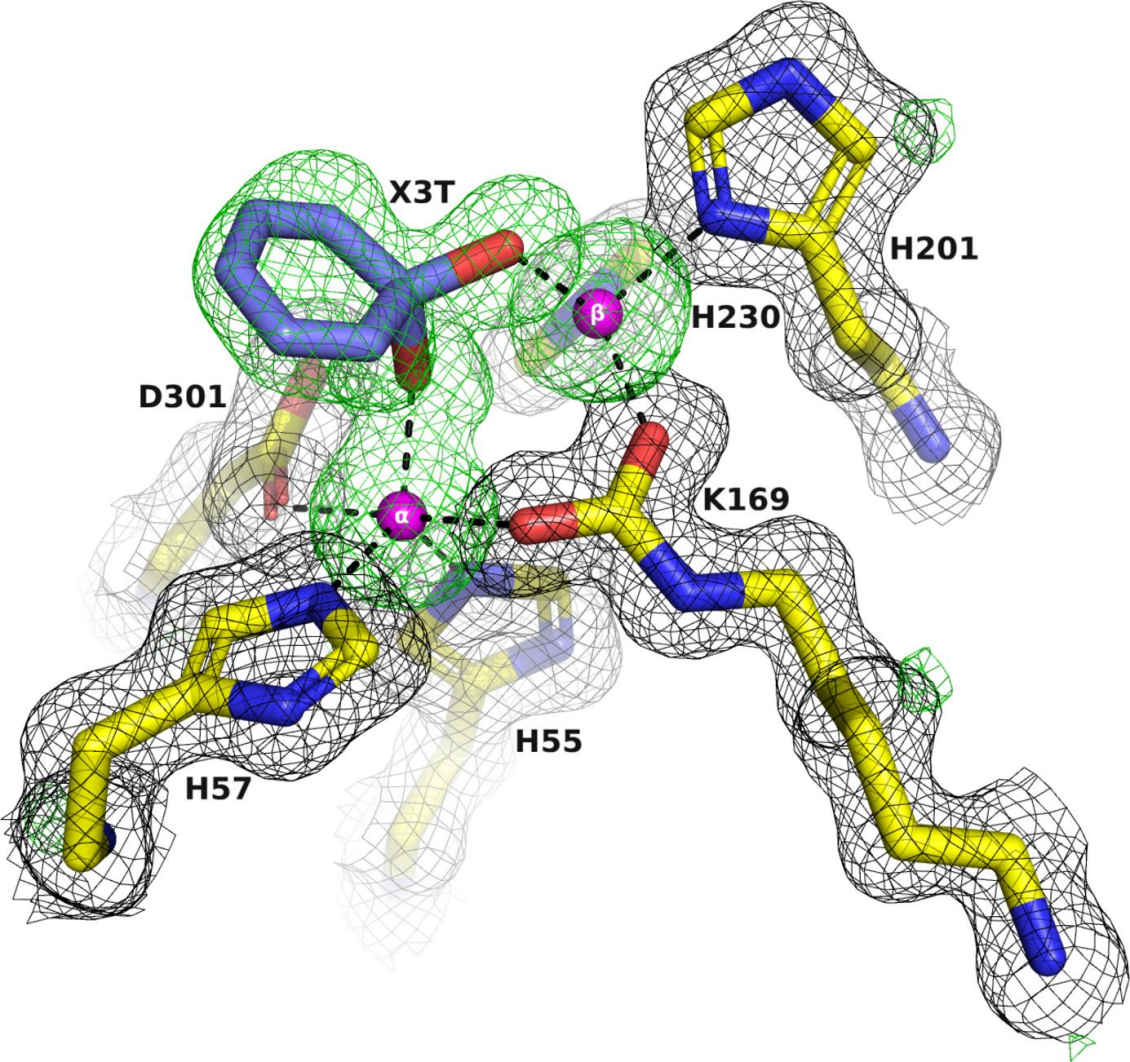
Electron-density omit map of the active-site region of C23M_1. The two Zn^2+^ ions and the electron density of an unidentified six-membered ring ligand, labeled X3T, were omitted in calculating the electron densities. The 2*F*_o_ − *F*_c_ omit map, contoured at 1σ, is shown in black. The *F*_o_ − *F*_c_ omit map, contoured at 3σ, is shown in green. The two Zn^2+^ ions are shown as magenta spheres.

**Figure 10:**
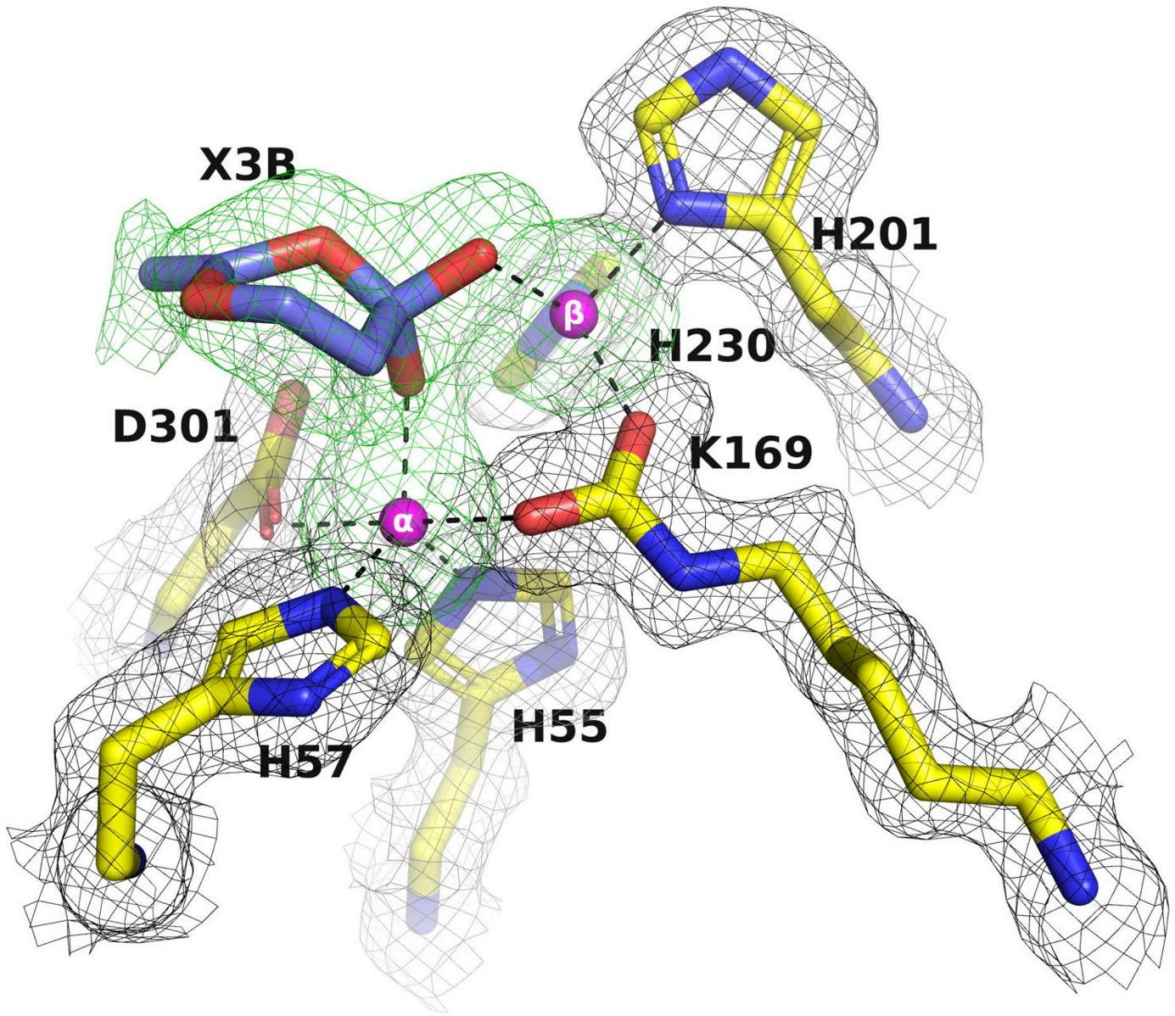
Electron-density omit map of the active-site region of A53_2. The two Zn^2+^ ions and the electron density of an unidentified six-membered ring, labeled X3B, were omitted in calculating the electron densities. The 2*F*_o_ − *F*_c_ omit map, contoured at 1σ, is shown in black. The *F*_o_ − *F*_c_ omit map, contoured at 3σ, is shown in green. The two Zn^2+^ ions are shown as magenta spheres.

The apo structures of A53_2 and C23M_1, which were crystallized from AS and glycerol, also showed the presence of six-membered ring ligands: X3B (Fig. 10 ▸) and X3T (Fig. 9 ▸), respectively. These ligands, similarly to X3E, act as chelators that coordinate the Zn^2+^ ions in the active site. It is therefore very likely that they were carried over from the protein expression and purification process. It is worth mentioning that in some published PTE structures, *i.e.* PDB entry 3a4j (Jackson, Foo *et al.*, 2009 ▸), water molecules were assigned within the active site. However, they also contain electron density in the active site that might correspond to unidentified six-membered rings similar to those that we observed.

Notably, when AS and glycerol are used as precipitants, the binding of OP ligands displaces the cyclic compounds X3B or X3T initially observed in the apo structures. This displacement and binding of the OPs will be discussed in more detail below.

### 3.7. Co-crystallization and soaking of OPs into PTEs

Soaking ligands into crystals is a common approach to obtain the structures of protein–ligand complexes. However, it requires the careful consideration of several factors, including the requirement to dissolve the ligand either in the crystallization precipitant or in a solvent that will not destroy the protein crystal, the anticipated soaking time and the choice of an effective ligand concentration. One significant limitation of soaking is the requirement for a crystal form with an accessible ligand-binding site or with a bound ligand that can easily be replaced by the ligand of interest. In the case of the PTE variants, attempts to soak the products of OP substrates into existing crystals of the native enzymes were unsuccessful because the active site was already occupied by ligands, such as the octapeptide spacer (Fig. 6 ▸), AA (Fig. 7 ▸) or the unidentified six-membered rings shown in Figs. 9 ▸ and 10 ▸, coordinated to the α-Zn^2+^ and β-Zn^2+^ ions, thus making it difficult for the soaked ligands to replace them.

Failure of the employed OP ligands to displace the ligands in the active site could be due to a lack of flexibility of the crystalline protein, to crystal-packing constraints or to the fact that the products may be bound more tightly than the incoming OP substrate. These limitations, taken together, contribute to the challenges faced when attempting to soak OP ligands into existing crystals of PTE variants. One way to overcome these challenges is to employ co-crystallization, which can facilitate ligand exchange within the active site in the absence of the constraints imposed by a pre-existing crystal structure.

**Figure 11:**
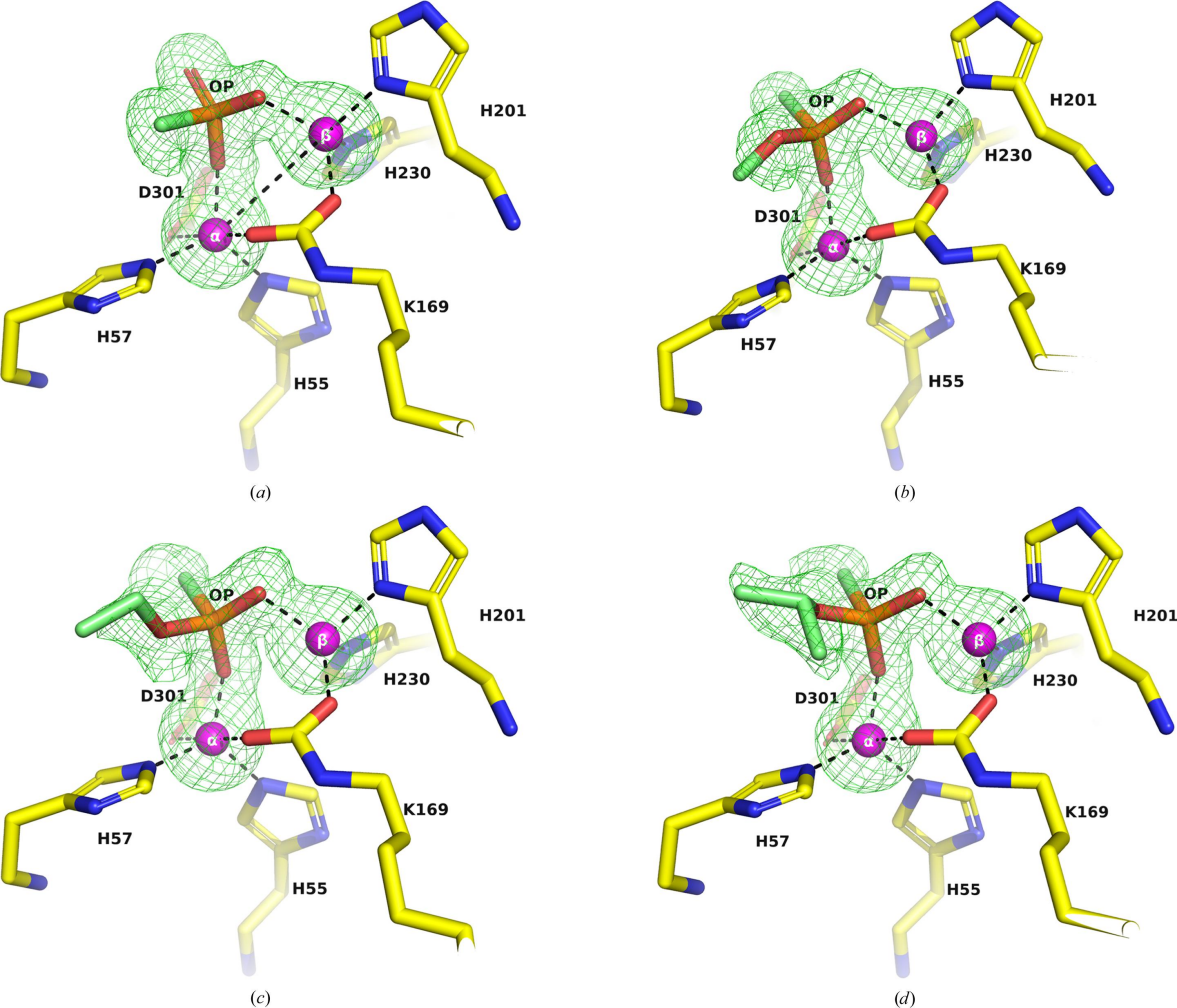
Electron-density omit maps (*F*_o_ − *F*_c_) of the active-site regions of PTEs. The two Zn^2+^ ions and the OPs were omitted from the calculations. (*a*) Methylphosphonate was observed in the C23M_2 structure. (*b*) *O*-Ethyl methylphosphonic acid was observed in C23_5. However, the electron density could only account for the first C atom of the OCH_2_CH_3_ substituent. (*c*) *O*-Ethyl methylphosphonic acid, the hydrolysis product of *O*-ethyl-*O*-(*N*,*N*-diisopropylaminoethyl) methylphosphonate, was observed in the C23_4 structure. (*d*) *O*-Isopropyl methylphosphonic acid was observed in C23_2. The interatomic distances observed between the O atoms of the P—O bond in all OP acid products are 1.9–2.0 Å. The *F*_o_ − *F*_c_ omit map, contoured at 3σ, is shown in green. The six residues that bind to the two Zn^2+^ ions are shown as stick representations, with C atoms colored yellow, N atoms blue, O atoms red and P atoms orange.

Co-crystallization is the method of choice when the ligands are insoluble in the crystallization precipitant, the crystal-packing constraints preclude soaking, ligand binding is associated with conformational changes or the active site is occupied by a ligand that cannot be displaced by the ligand of interest. Indeed, in the PTE variants studied, OP hydrolysis products could only be observed in the active site when co-crystallization was the method adopted, and only in crystals obtained from AS and glycerol in space group *P*4_3_2_1_2. The electron density in the active sites of the A53_5, C23_3 and C23M_2 structures co-crystallized with methylphosphonic acid (Fig. 11 ▸ *a*) clearly indicated that the ligand replaced the six-membered ring present in the apo structures (X3B in A53_2 and X3T in C23M_1), and two of the three O atoms of the OPs are in close contact with the two Zn^2+^ ions at similar interatomic distances of 1.9–2.0 Å. It is somewhat puzzling that in the complexes of methylphosphonic acid with the three PTE variants, A53, C23 and C23M, the methyl group of CH_3_P unequivocally projects in an identical direction, which clearly differs from that observed in the other OP conjugates (Figs. 11 ▸ *b*, 11 ▸ *c* and 11 ▸ *d*). In the case of co-crystallization with authentic *O*-ethyl methylphosphonic acid (C23_5; Fig. 11 ▸), the electron density observed could account only for the first C atom of the OCH_2_CH_3_ substituent. Since the second C atom would not make contact with any other amino-acid residue, it is likely to be disordered. In the case of the C23_4 structure co-crystallized with *O*-ethyl-*O*-(*N*,*N*-diisopropylaminoethyl) methylphosphonate (Fig. 3 ▸ *c*), the electron density is consistent with its hydrolysis product, *O*-ethyl methylphosphonic acid (Fig. 11 ▸ *b*). This suggested unexpected hydrolysis of *O*-ethyl-*O*-(*N*,*N*-diisopropylaminoethyl) methylphosphonate, either in the stored stock or in the crystallization medium. The rapid hydrolysis of this compound will be described elsewhere. The presence of a bound disordered moiety of detached *N*,*N*-diisopropylaminoethanol cannot be excluded.

The dissociation constant generated in this study for the reversible complex between wt PTE and authentic *O*-ethyl methylphosphonic acid (*K*_i_ = 4.3 m*M*) is eightfold greater than that obtained for methylphosphonic acid (*K*_i_ = 0.54 m*M*). Both are poor inhibitors of PTE when paraoxon is the substrate. The enhanced affinity of methylphosphonic acid is attributed to the greater density of negative charge due to the third P—O bond. This confirms the contribution of the negative charge density to association with the PTE binuclear center. The *O*-isopropyl methylphosphonic acid in C23_2 (Fig. 11 ▸ *d*) is oriented similarly to *O*-ethyl methylphosphonic acid in the active sites of C23_5 and C23_4 (Figs. 11 ▸ *b* and 11 ▸ *c*, respectively). Thus, based on visualization of the projections of different product ligands, in C23_2, C23_4 and C23_5 the following residues accommodate the P–O–ethyl and P–O–isopropyl moieties following detachment of the leaving group: His57, Gly60, Ile106, Trp131, Asp301, Leu303, Phe306 and Ser308. The CH_3_ group of the CH_3_P moiety in these OP ligands is projected into a space defined by His230, His257, Leu271, Asp301 and Phe306, an observation consistent with the previous report using the nonhydrolysable ligand *O*,*O*-diisopropyl methylphosphonate (Benning *et al.*, 2000 ▸). However, in the case of the three crystal structures of methylphosphonic acid (A53-5, C23-3 and C23M-2) the same methyl group is oriented in a different direction, into a space defined by His57, Ile106, Trp131, Asp301 and Leu303. The multiple orientations observed in the crystal structures of a variety of substituents attached to the P atom of either non­hydrolysable OPs or acid products of OP substrates can explain the promiscuity of PTEs.

Earlier studies suggested that the phosphonyl O atoms of *O*,*O*-diethyl 4-methylbenzylphosphonate (PDB entry 1dpm; Table 1 ▸; Vanhooke *et al.*, 1996 ▸) and diisopropyl methylphosphonate (PDB entry 1ez2; Table 1 ▸; Benning *et al.*, 2000 ▸), in their wt PTE complexes, interacted with the more exposed β-Zn^2+^ ion at distances of 3.5 and 2.5 Å, respectively, thus assigning it as the catalytic Zn^2+^. Notably, in both structures the phosphonyl O atoms were observed to be at distances of 4.7 and 5.0 Å, respectively, from the buried α-Zn^2+^ ion. The experimentally determined 3D structures of the OP acid products observed in the present study are consistent with the reported 3D structures of the complexes of PTEs with two other acid products: *O*,*O*-diethylphosphoric acid (DEP; PDB entry 3cak; Table 1 ▸; Kim *et al.*, 2008 ▸) and ethyl-4-methyl­benzylphosphonate (mEBP; PDB entry 7p85; Table 1 ▸; Job *et al.*, 2023 ▸). The P—O O atoms of PTE–DEP and PTE–mEBP are placed symmetrically at 2.0–2.2 Å away from the two Zn^2+^ ions, whereas in the case of the complex of the triester substrate analogue (C_2_H_5_O)_2_P(O)CH_2_phenyl-pCH_3_ the P=O O atom is 3.5 and 4.7 Å from the exposed β-Zn^2+^ and the buried α-Zn^2+^, respectively (Vanhooke *et al.*, 1996 ▸). These observations are consistent with the flexibility of the PTE active site, which results in a broad specificity.

The short distance between the P—O O atom of the acid products and the buried α-Zn^2+^ in the C23_2, C23_4 and C23_5 structures, together with the similar short distances of the P—O O atoms observed for *O*,*O*-diethylphosphoric acid (Kim *et al.*, 2008 ▸) and *O*-ethyl-4-methylbenzylphosphonic acid (Job *et al.*, 2023 ▸), suggests the involvement of both Zn^2+^ ions in catalysis.

## 4. Discussion

This study describes the challenges encountered in obtaining crystal structures of complexes of OP ligands with the enzyme PTE. Crystallization solutions contain a spectrum of chemicals that act as protein precipitants, buffers and/or reagents to increase protein stability. The effective metal concentration in solution may be substantially decreased by the formation of metal complexes with some of these chemicals. Some crystallization reagents or compounds used in the expression and purification processes may also act as chelators that coordinate metal ions. Therefore, it is essential to carefully consider the choice and concentration of reagents used in the pipeline from expression to crystallization so as to ensure that they do not perturb the functional state of the target protein (Newman, 2004 ▸).

Zn^2+^ ions play a crucial role in the activity of more than 300 enzymes, including PTE (McCall *et al.*, 2000 ▸). In initial attempts to obtain PTE crystals suitable for soaking with OPs, we obtained crystals of the A53 variant, which proved to contain an inactive monomer with only one Zn^2+^ ion, corresponding to the α-Zn^2+^ ion, in its active site. In contrast, the active form of the enzyme is a dimer, containing buried α-Zn^2+^ and exposed β-Zn^2+^ ions in each subunit, which are essential both for stabilizing the structure of the enzyme and for facilitating its catalytic activity (Holden & Raushel, 2021 ▸). That the two metal ions seen in the crystal structures studied were indeed Zn^2+^ ions was confirmed by the anomalous difference maps of X-ray data collected at the zinc absorption edge. To avoid forming inactive PTE molecules containing fewer than two Zn^2+^ ions in the active site, it was crucial to add ZnCl_2_ during protein expression, purification and crystallization.

To increase the expression levels and solubility of PTE, maltose-binding protein (MBP) was introduced as a fusion partner and was removed by digestion with factor Xa prior to crystallization trials. Unexpectedly, electron density was observed that could be ascribed to penetration of the PTE active site by the residual octapeptide spacer. The distance between the active site and the N-terminus of the octapeptide was approximately 4.5 Å. Notably, the residual octapeptide was observed in PTE crystals obtained from several crystallization conditions, all of which crystallized exclusively in space group *P*2_1_. The presence of the octapeptide in the active site might be expected to interfere with the binding of OPs. Thus, it is clear that the space group in which a protein crystallizes can affect the conformation of the protein and the binding of ligands. This is because the space group determines the crystal packing, which in turn affects the local environment around the protein molecules. It is worth noting that the space group in which a protein crystallizes is not typically taken into account in the computer-aided drug-design methods used to identify promising drug candidates, which rely on the protein structure alone to predict binding. Furthermore, our results highlight the importance of removing residual tags used to increase expression and purification levels before attempting to study the 3D structures of complexes and conjugates of proteins. In fact, when one overlays the recently determined structure of PTE complexed with ethyl-4-methylbenzylphos­phonate (mEBP; PDB entry 7p85; Table 1 ▸; Job *et al.*, 2023 ▸) on that of A53_3, the benzyl group of mEBP is seen to be very near the side chain of Ile26 of the octapeptide tag. This is strong supporting evidence that the octapeptide binds in a position similar to that of the large leaving group of the OP.

In the present study, it was observed that some of the crystallization precipitants and compounds used in the expression and purification processes could lodge within the active site of PTE. Specifically, the AA monomer of the PAA used as a precipitant was detected in the active site of the PTE variants, and well defined electron density corresponding to unidentified six-membered ring compounds was observed in the PTE active site when either PEG 6000 together with MPD, or AS together with glycerol, were employed in crystallization trials. Soaking OPs into crystals of the apo PTE variants grown from the three different crystallization conditions failed to replace either the six-membered ring compounds or the AA observed in the active site. We overcame this limitation by adapting co-crystallization protocols. We thus co-crystallized the A53, C23 or C23M variants in the presence of OPs under the same conditions used to crystallize the apo forms. However, in the co-crystallization experiments where either PEG 6000 together with MPD or PAA were used as crystallization conditions, the six-membered ring (X3E) and AA, respectively, also lodged within the active site of PTE and impeded OP binding. In the crystals obtained in space group *P*2_1_ the active site also contains the residual octapeptide tag used for expression, which is only observed in this space group. This octapeptide behaves like a peptide-inhibitor mimic and thus may help to define the mode of binding of substrates with an aliphatic leaving group.

Only when the PTE variants and the OPs are co-crystallized from AS with glycerol in space group *P*4_3_2_1_2 can the OP ligands displace the cyclic compounds X3B or X3T that were initially observed in the apo structures. Thus, electron density corresponding to methylphosphonate was observed in the active sites of the three PTE variants A53, C23 and C23M (see Table 2 ▸). The electron-density map indicates that the methyl group (CH_3_) of the methylphosphonate moiety unequivocally projects in an identical direction across all three variants. The *O*-ethyl methylphosphonate and *O*-isopropyl methylphosphonate moieties were observed in the active sites of the C23 structures (see Table 2 ▸). Electron density corresponding to *O*-ethyl methylphosphonate, the less expected hydrolysis product of *O*-ethyl-*O*-(*N*,*N*-diisopropylaminoethyl) methylphosphonate, was observed in the active site of the C23 structure, implying rapid hydrolysis of this ligand in aqueous solutions. The isopropyl group of *O*-isopropyl methylphos­phonate and the ethyl group of *O*-ethyl methylphosphonate project in the same direction, which clearly differs from that observed for the methyl group of methylphosphonate. The results obtained in the co-crystallization experiments in AS with glycerol in space group *P*4_3_2_1_2 defined an *O*-alkyl binding pocket.

**Figure 12:**
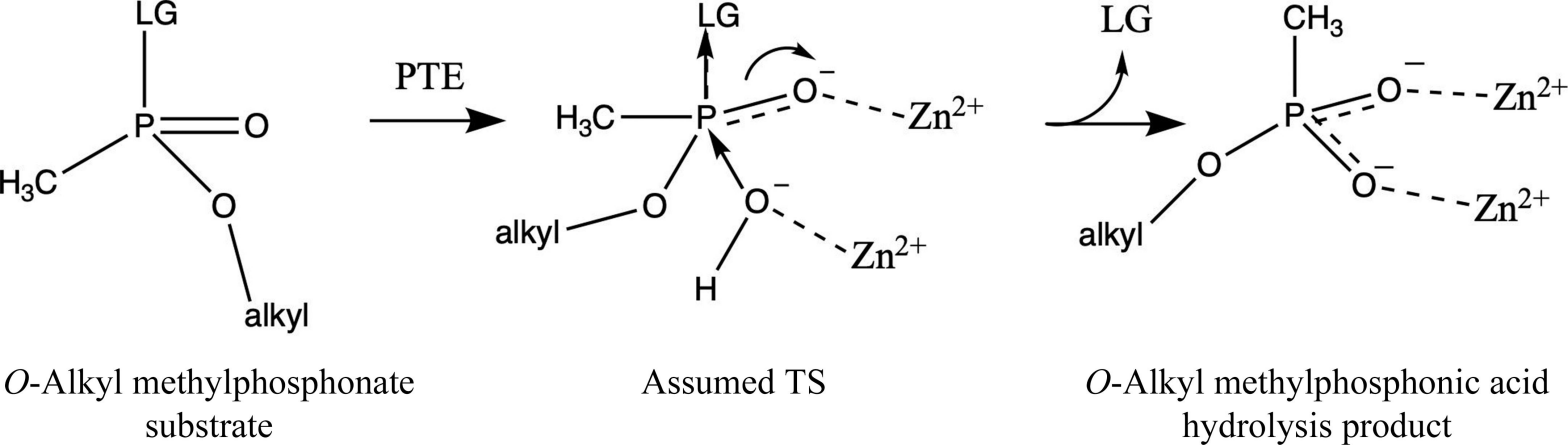
Schematic depiction of the putative TS for the hydrolysis of an *O*-alkyl methylphosphonate. The nucleophile is a hydroxide ion that originates from a water molecule that bridges the two Zn^2+^ ions. LG represents the leaving group. The geometries displayed are for the purposes of illustration and discussion.

The accepted catalytic mechanism for the hydrolysis of OP triesters by PTEs is based on data that are consistent with an S_N_2-type nucleophilic displacement of the leaving group by direct attack at the P atom (Bigley & Raushel, 2013 ▸; Koca *et al.*, 2001 ▸). The nucleophile is believed to be a water molecule (or hydroxide ion) that is clearly seen as well defined electron density bridging the two Zn^2+^ ions in a high-resolution PTE structure (PDB entry 2ob3). Thus, in all likelihood, the first step is the formation of a pentacoordinated transition state (TS) that develops two partially charged P—O bonds, as shown below schematically for the hydrolysis of an *O*-alkyl methylphosphonate with a suitable leaving group (LG) (Fig. 12 ▸).

The findings presented in this study may help to rationalize the role of the active-site metal center in PTEs. The OP products observed in the C23 crystal structures (Fig. 11 ▸) are in their ground state. Yet, they mimic the putative charged TS for the hydrolysis of *O*-alkyl methylphosphonates by offering two P—O bonds that ligate the two Zn^2+^ ions in a bidentate mode at interatomic distances of 1.9–2.0 Å (Fig. 11 ▸). Together with the similar short distances (2.0–2.2 Å) reported for PTE complexes with acidic OP products such as *O*,*O*-diethylphos­phoric acid (Kim *et al.*, 2008 ▸) and *O*-ethyl-4-benzylphosphonic acid (Job *et al.*, 2023 ▸), it is suggested that, regardless of the size of the substituent, the enzyme is sufficiently flexible to utilize the stabilization machinery offered by the two Zn^2+^ ions. Accordingly, the buried and exposed Zn^2+^ ions are proposed to stabilize the developing high-energy charged TS, thereby lowering its energy content, with concomitant acceleration of the reaction relative to water alone. A similar possibility was suggested in the case of a Zn^2+^-containing carbonic anhydrase in which a negatively charged transition state was envisaged (Christianson & Cox, 1999 ▸). The bimetal active-site centers formed by the Zn^2+^ ions seem to play a major role not only in polarizing the P=O bond and making the P atom more susceptible to nucleophilic attack, but also by stabilizing the TS through an electrostatic contribution, which is consistent with its key role in enzymatic catalysis (Warshel *et al.*, 2006 ▸). The rapid departure of the acid OP product guarantees the high turnover of OP substrates that in the case of paraoxon, for example, approach diffusion control of the reaction rate. Indeed, despite the the tight coordination of the acid OP product in the solid state, the dissociation constant *K*_i_ of 4.3 m*M* observed for *O*-ethyl methylphosphonic acid reveals poor affinity for PTE in aqueous solution. Similarly, analysis of the crystal structure of the complex of the P=S-containing product of dimethoate hydrolysis, dimethylthiophosphate, suggested that the ∼1000-fold slower turnover of the phosphorothiolates by phosphotriesters when compared with the P=O homologues may be attributed to slow departure of the bound product seen in the bimetal catalytic center (Jackson *et al.*, 2005 ▸). However, further experiments are required to substantiate this contention.

Our study highlights the significant influence of several factors on the successful crystallization of protein–ligand complexes. These factors include the molecular constructs used, the presence of residual tags, the choice of space groups, the compounds employed for crystallization and the compounds carried over from protein expression and purification processes. In the case of PTE, we observed that these compounds could occupy the active site, effectively competing with, or even preventing, the binding of OPs, which are of particular interest for drug development. This can potentially lead to the misidentification of lead drug candidates. By considering and by constructively addressing these factors, one can enhance the likelihood of obtaining crystals of protein–ligand complexes that faithfully represent the desired interaction, allowing more accurate and reliable characterization of ligand binding and aiding in the rational design of potential therapeutic agents.

## Supplementary Material

PDB reference: A53_1, 8p7f

PDB reference: A53_2, 8p7h

PDB reference: A53_3, 8p7i

PDB reference: A53_4, 8p7k

PDB reference: A53_5, 8p7m

PDB reference: C23_1, 8p7n

PDB reference: C23_2, 8p7q

PDB reference: C23_3, 8p7r

PDB reference: C23_4, 8p7s

PDB reference: C23_5, 8p7t

PDB reference: C23M_1, 8p7u

PDB reference: C23M_2, 8p7v

## Figures and Tables

**Table 1:** Crystal structures of PTEs

No.	PDB code	Ligands in the active site	Resolution (Å)	Reference
Apo				
1	4xaz	Zn^2+^	1.55	Campbell *et al.* (2016 ▸)
2	4pcn	Zn^2+^	1.54	Campbell *et al.* (2016 ▸)
3	4zst	Co^2+^	2.01	Bigley *et al.* (2015 ▸)
4	4zsu	Co^2+^	2.01	Bigley *et al.* (2015 ▸)
5	3upm	Co^2+^	1.95	Tsai *et al.* (2012 ▸)
6	3ura	Co^2+^	1.88	Tsai *et al.* (2012 ▸)
7	2ob3	Zn^2+^	1.04	J. Kim, U. A. Ramagopal, P. Tsai, F. M. Raushel & S. C. Almo (unpublished work)
8	1pta	No metal ion	2.1	Benning *et al.* (1994 ▸)
9	1p6b	Zn^2+^	1.9	Hill *et al.* (2003 ▸)
10	4gy0	Zn^2+^	1.85	Tokuriki *et al.* (2012 ▸)
11	6bhk	Zn^2+^	1.4	C. M. Miton, E. C. Campbell, C. J. Jackson & N. Tokuriki (unpublished work)
12	3a4j	Co^2+^	1.25	Jackson, Foo *et al.* (2009 ▸)
13	3a3x	Co^2+^	1.7	Jackson, Foo *et al.* (2009 ▸)
14	6gbk	Zn^2+^	1.9	Khersonsky *et al.* (2018 ▸)
15	6gbj	Zn^2+^	1.63	Khersonsky *et al.* (2018 ▸)
16	3wml	Co^2+^ and Fe^2+^	1.63	Khersonsky *et al.* (2018 ▸)
17	5w7h	Zn^2+^	2.75	Campbell *et al.* (2018 ▸)
18	5vej	Co^2+^	1.3	Selleck *et al.* (2017 ▸)
With cacodylate/ethylene glycol/glycerol in the active site				
1	4xd3	Zn^2+^ and cacodylate	1.57	Campbell *et al.* (2016 ▸)
2	4xd4	Zn^2+^ and cacodylate	1.9	Campbell *et al.* (2016 ▸)
3	4xd5	Zn^2+^ and cacodylate	1.85	Campbell *et al.* (2016 ▸)
4	4xd6	Zn^2+^ and cacodylate	1.75	Campbell *et al.* (2016 ▸)
5	4xaf	Zn^2+^ and cacodylate	1.66	Campbell *et al.* (2016 ▸)
6	4xag	Zn^2+^ and cacodylate	1.6	Campbell *et al.* (2016 ▸)
7	4xay	Zn^2+^ and cacodylate	1.84	Campbell *et al.* (2016 ▸)
8	4pbe	Zn^2+^ and cacodylate	1.51	Campbell *et al.* (2016 ▸)
9	4pbf	Zn^2+^ and cacodylate	1.9	Campbell *et al.* (2016 ▸)
10	4pcp	Zn^2+^ and cacodylate	1.63	Campbell *et al.* (2016 ▸)
11	3ur2	Co^2+^ and imidazole	2.0	Tsai *et al.* (2012 ▸)
12	3cs2	Co^2+^ and cacodylate	1.95	Kim *et al.* (2008 ▸)
13	2oql	Zn^2+^ and glycerol	1.8	J. Kim, F. M. Raushel & S. C. Almo (unpublished work)
14	2o4m	Zn^2+^ and cacodylate	1.64	J. Kim, U. A. Ramagopal, P. Tsai, F. M. Raushel & S. C. Almo (unpublished work)
15	2o4q	Zn^2+^ and cacodylate	1.95	Kim *et al.* (2008 ▸)
16	4gy1	Zn^2+^ and cacodylate	1.5	Tokuriki *et al.* (2012 ▸)
17	1jgm	Cd^2+^ and ethylene glycol	1.3	Benning *et al.* (2001 ▸)
18	1hzy	Zn^2+^ and ethylene glycol	1.3	Benning *et al.* (2001 ▸)
19	2d2j	Co^2+^ and ethylene glycol	1.75	Jackson *et al.* (2005 ▸)
20	1i0b	Mn^2+^ and ethylene glycol	1.3	Benning *et al.* (2001 ▸)
21	5wcw	Zn^2+^ and cacodylate	1.46	C. M. Miton, E. C. Campbell, C. J. Jackson & N. Tokuriki (unpublished work)
22	5wj0	Zn^2+^ and cacodylate	1.65	C. M. Miton, E. C. Campbell, C. J. Jackson & N. Tokuriki (unpublished work)
23	5wiz	Zn^2+^ and cacodylate	1.96	C. M. Miton, E. C. Campbell, C. J. Jackson & N. Tokuriki (unpublished work)
24	5wcr	Zn^2+^ and cacodylate	1.75	C. M. Miton, E. C. Campbell, C. J. Jackson & N. Tokuriki (unpublished work)
25	5wcp	Zn^2+^ and cacodylate	1.50	C. M. Miton, E. C. Campbell, C. J. Jackson & N. Tokuriki (unpublished work)
26	5wms	Zn^2+^ and cacodylate	1.60	C. M. Miton, E. C. Campbell, C. J. Jackson & N. Tokuriki (unpublished work)
27	6bhl	Zn^2+^ and cacodylate	1.40	C. M. Miton, E. C. Campbell, C. J. Jackson & N. Tokuriki (unpublished work)
28	1i0d	Zn^2+^, Cd^2+^ and ethylene glycol	1.3	Benning *et al.* (2001 ▸)
29	6aml	Zn^2+^ and cacodylate	1.46	C. M. Miton, E. C. Campbell, C. J. Jackson & N. Tokuriki (unpublished work)
30	3oqe	Co^2+^ and ethylene glycol	1.9	Ely *et al.* (2010 ▸)
31	6bh7	Zn^2+^ and cacodylate	1.40	C. M. Miton, E. C. Campbell, C. J. Jackson & N. Tokuriki (unpublished work)
32	6gbl	Zn^2+^ and cacodylate	1.95	Khersonsky *et al.* (2018 ▸)
With substrate analogues/inhibitors in the active site				
1	4e3t	Hexyl(naphthalene-2-yloxy)phosphinic acid (in one monomer)	1.65	Tokuriki *et al.* (2012 ▸)
2	6b2f	Hexyl(naphthalene-2-yloxy)phosphinic acid	1.77	Tokuriki *et al.* (2012 ▸)
3	3ur5	Co^2+^ and diethylphosphate	1.6	Tsai *et al.* (2012 ▸)
4	3urb	Co^2+^ and diethylphosphate	1.77	Tsai *et al.* (2012 ▸)
5	3urn	Co^2+^ and cyclohexyl methylphosphonate	1.95	Tsai *et al.* (2012 ▸)
6	3urq	Co^2+^ and cyclohexyl methylphosphonate	2.1	Tsai *et al.* (2012 ▸)
7	3cak	Co^2+^ and diethyl hydrogen phosphate	1.83	Kim *et al.* (2008 ▸)
8	3e3h	Co^2+^ and diethyl 4-methylbenzylphosphonate	2.15	Reeves *et al.* (2008 ▸)
9	1qw7 ^^^	Co^2+^ and 4-methylbenzylphosphonate (not in active site)	1.9	Grimsley *et al.* (2005 ▸)
10	1eyw	Zn^2+^ and triethylphosphate	1.9	Benning *et al.* (2000 ▸)
11	1ez2	Zn^2+^ and diisopropyl methylphosphonate	1.9	Benning *et al.* (2000 ▸)
12	1dpm	Zn^2+^ and diethyl 4-methylbenzylphosphonate	2.1	Vanhooke *et al.* (1996 ▸)
13	2d2g	Co^2+^ and dimethyl hydrogen thiophosphate	1.85	Jackson *et al.* (2005 ▸)
14	2d2h	Co^2+^ and trimethylphosphate	1.8	Jackson *et al.* (2005 ▸)
15	3so7	Co^2+^ and phosphate ion	2.2	Ely *et al.* (2012 ▸)
16	3c86	Co^2+^ and diethyl hydrogen thiophosphate	1.8	Jackson *et al.* (2008 ▸)
17	1psc	Cd^2+^ and diethyl 4-methylbenzylphosphonate (not in active site)	2.0	Benning *et al.* (1995 ▸)
18	2r1m	Fe^2+^, Co^2+^ and diethyl hydrogen phosphate	1.55	Jackson *et al.* (2008 ▸)
19	2r1n	Fe^2+^, Co^2+^ and diethyl 4-methylbenzylphosphonate	1.7	Jackson *et al.* (2008 ▸)
20	2r1k	Fe^2+^, Co^2+^ and diethyl hydrogen phosphate	2.1	Jackson *et al.* (2008 ▸)
21	2r1l	Fe^2+^, Co^2+^ and *O*,*O*-diethyl hydrogen triphosphate	1.95	Naqvi *et al.* (2014 ▸)
22	3ood	Co^2+^ and diethyl 4-methylbenzylphosphate	1.89	Ely *et al.* (2010 ▸)
23	4np7	Fe^2+^, Co^2+^ and *O*,*O*-diethyl hydrogen triphosphate	1.99	Ely *et al.* (2010 ▸)
24	7p85	Zn^2+^ and ethyl 4-methylbenzylphosphonate	1.47	Job *et al.* (2023 ▸)
25	3a3w	Co^2+^ and diethyl 4-methylbenzylphosphate	1.85	Jackson, Foo *et al.* (2009 ▸)

**Table:** Values in parentheses are for the highest resolution shell.

	A53_1	A53_2	A53_3	A53_4	A53_5	C23_1
Ligand in the active site	Apo, 1 Zn^+2^	Apo, X3B	Apo, X3E	Apo, polyacrylic acid	Methylphosphonate	Apo
Crystallization conditions	12% PEG 6000, 5% MPD, 0.1 *M* HEPES pH 7.5	1.5 *M* (NH_4_)_2_SO_4_, 17% glycerol, 0.1 *M* Tris pH 7.5	16% PEG 6000, 2% MPD, 0.1 *M* HEPES pH 7.5, 0.1 *M* glycine	22% polyacrylic acid, 0.02 *M* MgCl_2_, 0.1 *M* HEPES pH 7.5	1.5 *M* (NH_4_)_2_SO_4_, 12% glycerol, 0.1 *M* Tris pH 7.5	10% PEG 6000, 2% PEG 400, 15% MPD, 0.1 *M* HEPES pH 7.5
Data collection						
Source	ID14-4, ESRF	Rigaku home source	Rigaku home source	Rigaku home source	Rigaku home source	ID14-4, ESRF
PDB code	8p7f	8p7h	8p7i	8p7k	8p7m	8p7n
Space group	*P*2_1_2_1_2	*P*4_3_2_1_2	*P*2_1_	*P*2_1_	*P*4_3_2_1_2	*P*2_1_2_1_2_1_
*a*, *b*, *c* (Å)	79.70, 93.67, 44.59	69.67, 69.67, 186.83	54.23, 81.25, 70.66	55.03, 81.49, 70.81	69.74, 69.74, 186.76	88.89, 119.90, 161.49
α, β, γ (°)	90, 90, 90	90, 90, 90	90, 94.80, 90	90, 94.99, 90	90, 90, 90	90, 90, 90
Copies in asymmetric unit	1	1	2	2	1	4
Resolution (Å)	39.85–2.00	25.48–1.77	36.77–1.70	32.37–1.93	27.94–1.85	77.78–3.20
Outer shell (Å)	2.071–2.00	1.84–1.77	1.76–1.70	2.00–1.93	1.92–1.85	3.31–3.20
Unique reflections	23037 (2273)	44626 (4284)	67098 (6633)	46379 (4410)	39668 (3811)	29131 (2870)
Completeness (%)	98.91 (99.91)	97.93 (95.84)	99.69 (98.94)	98.69 (93.67)	98.24 (96.43)	99.61 (99.90)
Average *I*/σ(*I*)	12.50 (4.20)	22.90 (7.40)	20.06 (4.06)	10.80 (2.16)	36.19 (11.23)	7.24 (2.64)
Multiplicity	5.3 (5.5)	17.0 (16.7)	6.1 (5.8)	3.5 (2.9)	17.2 (17.0)	4.1 (4.2)
CC_1/2_	0.727 (0.638)	0.952 (0.910)	0.925 (0.925)	0.995 (0.772)	1 (0.989)	0.987 (0.823)
*R*_p.i.m._	0.218 (0.306)	0.086 (0.141)	0.024 (0.176)	0.052 (0.338)	0.014 (0.063)	0.09627 (0.3261)
Refinement						
Resolution range (Å)	39.85–2.00	25.48–1.77	36.77–1.70	32.37–1.93	27.94–1.85	71.41–3.20
Reflections [*I*/σ(*I*) > 0]	23010 (2271)	44611	67085	46363	39642	29063
Reflections in test set	2000 (197)	4283	3397	2355	3809	1412
*R*_work_/*R*_free_	0.2014/0.2297	0.1574/0.1894	0.1511/0.1808	0.1593/0.2119	0.1612/0.1876	0.1830/0.2546
Protein atoms	2163	2535	5067	5015	2506	9890
Water molecules	86	401	537	463	313	8
Overall average *B* factor (Å^2^)	41.93	20.23	25.16	27.32	18.52	44.15
Root-mean-square deviations						
Bond lengths (Å)	0.007	0.006	0.006	0.007	0.006	0.010
Bond angles (°)	0.85	0.85	0.85	0.83	0.92	1.21
Ramachandran plot						
Most favored (%)	97.50	96.94	97.58	97.25	97.24	95.69
Additionally allowed (%)	2.14	3.06	2.42	2.75	2.76	4.08
Disallowed (%)	0.36	0.00	0.00	0.00	0.00	0.23

**Table:** 

	C23_2	C23_3	C23_4	C23_5	C23M_1	C23M_2
Ligand in the active site	*O*-Isopropyl methylphosphonate	Methylphosphonate	*O*-Ethyl methylphosphonic acid	*O*-Ethyl methylphosphonate	Apo, X3T (zinc wavelength)	Methylphosphonate
Crystallization conditions	1.5 *M* (NH_4_)_2_SO_4_, 17% glycerol, 0.1 *M* Tris pH 7.0	1.5 *M* (NH_4_)_2_SO_4_, 17% glycerol, 0.1 *M* Tris pH 8.5	1.0 *M* (NH_4_)_2_SO_4_, 12% glycerol, 0.1 *M* Tris pH 7.5	1.5 *M* (NH_4_)_2_SO_4_, 17% glycerol, 0.1 *M* Tris pH 8.5	1.5 *M* (NH_4_)_2_SO_4_, 12% glycerol, 0.1 *M* Tris pH 8.0	1.5 *M* (NH_4_)_2_SO_4_, 12% glycerol, 0.1 *M* Tris pH 8.0
Data collection						
Source	Rigaku home source	Rigaku home source	Rigaku home source	Rigaku home source	ID23-1, ESRF	Rigaku home source
PDB code	8p7q	8p7r	8p7s	8p7t	8p7u	8p7v
Space group	*P*4_3_2_1_2	*P*4_3_2_1_2	*P*4_3_2_1_2	*P*4_3_2_1_2	*P*4_3_2_1_2	*P*4_3_2_1_2
*a*, *b*, *c* (Å)	69.56, 69.56, 186.64	69.83, 69.83, 186.78	69.54, 69.54, 185.84	69.45, 69.45, 186.97	69.69, 69.69, 187.07	69.70, 69.70, 186.48
α, β, γ (°)	90.0, 90.0, 90.0	90, 90, 90	90, 90, 90	90, 90, 90	90, 90, 90	90, 90, 90
Copies in asymmetric unit	1	1	1	1	1	1
Resolution (Å)	34.78–1.77	28.43–1.85	33.77–1.77	23.19–1.80	18.11–1.38	25.46–1.74
Outer shell (Å)	1.87–1.77	1.92–1.85	1.84–1.77	1.86–1.80	1.42–1.38	1.80–1.74
Unique reflections	45349 (4453)	40443 (3956)	45145 (4428)	42215 (4018)	96151 (9438)	47579 (4555)
Completeness (%)	99.92 (100.00)	99.94 (100.00)	99.89 (99.84)	97.23 (94.96)	99.15 (98.70)	98.25 (96.36)
Average *I*/σ(*I*)	18.97 (6.41)	33.96 (5.21)	30.97 (12.07)	22.59 (5.53)	6.87 (2.03)	20.05 (3.92)
Multiplicity	20.4 (20.6)	12.0 (11.7)	11.8 (11.4)	20.4 (20.6)	6.5 (6.4)	20.0 (20.0)
CC_1/2_	0.999 (0.974)	0.859 (0.689)	0.888 (0.914)	0.999 (0.948)	0.992 (0.776)	0.999 (0.895)
*R*_p.i.m._	0.0241 (0.0978)	0.1528 (0.2906)	0.09951 (0.1210)	0.02038 (0.1358)	0.05118 (0.2740)	0.02354 (0.2009)
Refinement						
Resolution range (Å)	31.11–1.77	28.43–1.85	31.10–1.77	22.97–1.80	18.11–1.38	24.88–1.74
Reflections [*I*/σ(*I*) > 0]	45340	40437	45111	42212	95540	47567
Reflections in test set	4453	2015	4428	4107	4684	2326
*R*_work_/*R*_free_	0.1670/0.1910	0.1548/0.1782	0.1602/0.1841	0.1569/0.1844	0.1572/0.1781	0.1668/0.1886
Protein atoms	2499	2541	2540	2522	2557	2506
Water molecules	259	377	346	268	372	305
Overall average *B* factor (Å^2^)	19.17	17.54	18.88	20.07	19.53	20.12
Root-mean-square deviations						
Bond lengths (Å)	0.016	0.006	0.006	0.006	0.066	0.007
Bond angles (°)	2.00	0.92	0.92	1.03	0.95	1.02
Ramachandran plot						
Most favored (%)	96.34	97.26	97.85	97.26	96.34	97.86
Additionally allowed (%)	3.35	2.74	2.15	2.74	3.66	1.83
Disallowed (%)	0.30	0.00	0.00	0.00	0.00	0.31
